# Climatic niche divergence drives patterns of diversification and richness among mammal families

**DOI:** 10.1038/s41598-018-27068-y

**Published:** 2018-06-08

**Authors:** Adrián Castro-Insua, Carola Gómez-Rodríguez, John J. Wiens, Andrés Baselga

**Affiliations:** 1 Departamento de Zoología, Facultad de Biología, Universidad de Santiago de Compostela, Rúa Lope Gómez de Marzoa, 15782 Santiago de Compostela, Spain; 2 Department of Ecology and Evolutionary Biology, University of Arizona, Tucson, AZ 85721-0088 USA

## Abstract

A major goal of evolutionary biology is to understand why clades differ dramatically in species richness. A key to this challenge is to uncover the correlates of variation in diversification rate (speciation – extinction) among clades. Here, we explore the relationship between diversification rates and the climatic niches of species and clades among 92 families of terrestrial mammals. We use a time-calibrated molecular phylogeny of mammals and climatic data from 3335 species. We show that considerable variation in net diversification rates among mammal families is explained by niche divergence (59%) and rates of niche change (51%). Diversification rates in turn explain most variation in species richness among families (79%). Contrary to expectations, patterns of diversification are not explained by differences in geographic range areas of clades, nor by their climatic niche position (i.e. whether they are primarily tropical or temperate). Overall, these results suggest that speciation through climatic niche divergence may help drive large-scale patterns of diversification and richness. Our results help explain diversification patterns in a major clade of vertebrates, and suggest that similar underlying principles may explain the diversification of many terrestrial clades.

## Introduction

A fundamental goal of evolutionary biology and related disciplines is to understand why some clades have more species than others^1^. In general, differences in current species richness between clades will be explained by either their age (e.g., older clades with more species) or by differences in how quickly they have accumulated species (i.e., net diversification rate). The net diversification rate of a clade is the outcome of speciation and extinction over time^2–4^. Differences in species richness among clades of the same rank (e.g., families, phyla) seem to be explained largely by differences in these net diversification rates^5^. Therefore, to understand richness patterns among clades, it is essential to uncover the ecological and evolutionary processes that determine differences in diversification rates among clades.

Climatic niches may be one of the key factors that drive variation in diversification rates and species richness among clades. Every terrestrial species has a realized climatic niche, which is the set of large-scale temperature and precipitation conditions where that species occurs^6–8^. The realized climatic niche can reflect physiological tolerances to these climatic conditions, but can also be influenced by other abiotic and biotic factors. The climatic niche of a species may strongly influence where it can occur over space and time^7^, and thus may be critically important for both speciation (e.g., ecological speciation through climatic niche divergence^9,10^), and extinction (e.g., due to climate change). We can thus expect a relationship between patterns of variation in climatic niches across clades and their net diversification rates. In support of this idea, some studies have shown that the climatic regime where a clade occurs can influence its diversification rate (e.g., faster diversification in tropical clades^11^). Similarly, other studies have shown that the rate of change in climatic niches among species within a clade can be positively related to species diversification^12–16^.

Differences in climatic niche width among species and clades may also influence diversification rates. Climatic niche width is the range of climatic conditions where a species or clade occurs (e.g., maximum yearly temperature – minimum yearly temperature across localities), for one or more climatic niche variables, instead of the values of the variables themselves. Gómez-Rodríguez *et al*.^17^ developed a framework to understand how climatic niche widths of species and clades might be related to diversification (and why). They outlined five competing hypotheses. First, the null hypothesis (H0) is that faster diversification rates lead to higher richness and higher richness leads to wider clade niches, but only because a clade with more species should span more divergent climatic conditions due to sampling alone, all else being equal. This hypothesis predicts a relationship between diversification rates and family-level niche width, but also predicts that the levels of climatic niche divergence can be explained by sampling size alone (i.e. number of species). Second, if speciation occurs mostly due to climatic niche conservatism, a negative correlation between clade-level diversification rates and niche width is expected (H1). In this scenario, a narrower species niche would lead to more fragmentation of species ranges when barriers of climatically unsuitable habitats appear, and thus more opportunities for allopatric speciation^18^. Alternatively, if speciation is predominantly linked to climatic niche divergence^9,10^, a positive relationship between diversification rate and clade-level niche width is expected (H2). Another hypothesis (H3) suggests that a positive relationship between diversification rate and clade-level niche width could arise from reduced extinction rates in clades with wider species-level niches that buffer them from the negative impacts of climatic fluctuations. This hypothesis minimally predicts a relationship between diversification rates and species-level niche widths. Alternatively (H4), wider family-level niche width could be indirectly related to diversification rate via a positive relationship between family-level niche width and the geographic extent of clades. Under this hypothesis, a larger geographic range area for a clade could lead to a larger range of climatic conditions experienced by its species, and the larger range area could more directly increase diversification rate by increasing the chances of allopatric speciation, or diminishing the probability of extinction^19–22^. This hypothesis predicts a relationship between diversification and family-level niche widths, but also predicts that there will be little unique effect of niche width when the area of each clade is accounted for statistically. Finally (H5), a relationship between diversification rate and niche width could also be the result of coincident geographic patterns of clade niche width and diversification rate. For example, tropical clades could be characterized both by narrower species-level niches for temperature-related variables^23,24^ and faster diversification rates than temperate clades, but with no causal relationship between niche widths and diversification rates.

Gómez-Rodríguez *et al*.^17^ tested these hypotheses using climatic, distributional, and phylogenetic data from amphibian species and families. They found that diversification rates of families were strongly and positively related to family-level niche widths, but weakly related to mean species niche widths of families. Additionally, the variable that explained the most variance in diversification rates of amphibian families (53%) was an indirect measure of niche divergence (i.e. families with narrow species-level niche widths but wide family-level niche widths). Taken together, the results were consistent with the hypothesis that diversification rates were strongly influenced by speciation linked to climatic niche divergence (H2).

In that study, niche divergence was quantified in absolute terms (i.e. present day differences in climatic niches between species in clades), ignoring the evolutionary rate of change. However, if niche divergence is linked to speciation and hence to diversification rate, then the rate of niche divergence over time should be associated with diversification rate even more strongly than with absolute niche divergence. Previous studies have tested the relationship between rates of climatic niche evolution and diversification in several groups, including the Cape flora^13^, plethodontid salamanders^12^, frogs^16^, all amphibians^25^, and birds^14,15,26^. A positive relationship was generally supported (but not across amphibians^25^). Likewise, absolute niche divergence was the strongest correlate of variation in diversification rate among amphibian families^17^. However, to our knowledge, no study has assessed the relative importance of absolute niche divergence and rates of niche evolution in explaining variation in diversification rates among clades.

Here we perform such an analysis across mammals. Our results shed light on the ecological factors linked to patterns of macroevolution and species richness in a major clade of vertebrates. They also address whether clade diversification is more strongly related to the absolute differences in the environmental conditions occupied by the species in a clade or to the velocity with which they occupy different environmental conditions, and how these latter two variables are related.

We analyse diversification rates in mammal families (dependent variable), and their relationship to climatic niche width, niche divergence, and rate of niche evolution (independent variables). Previous studies have addressed some related issues in mammals, but not the specific questions we address here. For example, Cooper *et al*.^27^ examined patterns of climatic niche evolution in mammals, and found that tropical, small-ranged, and specialized mammals tended to have slower rates of thermal-niche evolution than temperate, large-ranged, and generalist mammals (see also Cadena *et al*.^28^). However, they did not relate climatic niche evolution to patterns of diversification. Past studies showed that diversification rates in mammals are related to geographic or life-history factors (e.g., in Australian mammals^21^), trophic strategy^29^, and climatic niche width^30^, but did not address how much variation in diversification rates across mammals is explained by these variables. Latitudinal patterns in diversification rates among mammalian species and clades were also assessed in previous analyses, with the goal of explaining higher tropical species richness. These studies found higher diversification rates in tropical lineages at broad phylogenetic scales^11^, but not among genera^31^ or species^32^. However, they did not test if tropical distribution helps explain patterns of diversification and richness among mammalian families. Other studies have addressed patterns of diversification over time across all mammals^33–35^ or at lower taxonomic levels^36–39^, but did not focus on the correlates of diversification rates or richness among clades.

Here we test the relationships between niche evolution and diversification using climatic, distributional, and phylogenetic data for 3335 terrestrial mammal species in 92 families. We explore the relationships between diversification rates and: (i) average species niche widths within clades (families), (ii) clade-level niche widths (range of climatic conditions across all species within a family), (iii) absolute niche divergence among species within clades, (iv) rates of niche evolution within clades, (v) the mean position of the clade’s niche (e.g., tropical vs. temperate), and (vi) the geographic range area of the clade. Note that we treat diversification rate as the dependent variable, and therefore describe how much variation in diversification rate is explained by other variables (following standard statistical terminology). Nevertheless, we are initially neutral about the direction of causality underlying the relationship between niche divergence and diversification. However, by testing these alternate hypotheses we can gain insights on the causality and mechanisms involved (see Discussion).

## Materials and Methods

### Climatic niche width

All range maps for terrestrial mammal species available from the IUCN database^40^ were downloaded (*n* = 5285). This GIS database basically covers all described mammal species (5488 known species, including marine species). By “terrestrial” we mean non-marine, and thus marine species and families were excluded (given that we lack climatic data for these species). Species climatic niches were estimated from their distributions, as the climatic conditions in which each species lives (a Grinnellian niche operationally estimated following Peterson *et al*.^41^). Specifically, climatic data at 2.5 arc-minutes (or approximately 4.5 km) resolution were downloaded from the WorldClim database^42^, and niches were estimated by extracting the climatic values in the geographic range of a species or clade using the package raster^43^ in R^44^. Details on the functions used are given in Appendix S1 in the Supplementary Material. Six variables were selected to represent the climatic niche of mammal species, following standard practice in similar studies^17,24^: annual mean temperature (BIO1), maximum temperature of the warmest month (BIO5), minimum temperature of the coldest month (BIO6), annual precipitation (BIO12), precipitation of the wettest quarter (BIO16), and precipitation of the driest quarter (BIO17). These variables represent annual means and extreme values of temperature and precipitation, so it is assumed that they can give a robust description of species’ climatic niches. Other climatic variables in the WorldClim dataset generally represent minor variations on these six.

For each species, the climatic niche width was calculated following Gómez-Rodríguez *et al*.^17^. The niche width for each variable and species was defined as the difference between the minimum and maximum values across the species’ geographic range (extracted for each grid cell). The overall climatic niche width for each species was then the product of the species’ climatic ranges (niche widths) multiplied across the six variables. The climatic niche could not be estimated for three species having very small geographic ranges in areas with no climatic data available. To allow the inclusion of variables in different units (temperature and precipitation) in niche-width estimation, niche widths for each species for each climatic niche variable were standardized considering the maximum and minimum values for that variable across all species. Thus, for each species *i* in a set of *j* species, the standardized climatic range (niche width) is:

$${{\rm{StRg}}}_{{\rm{i}}}={[\mathrm{Rg}}_{{\rm{i}}}-{\min (\mathrm{Rg}}_{{\rm{1}}}{:\mathrm{Rg}}_{j)}]/[\,\max ({{\rm{Rg}}}_{1}\,{:\mathrm{Rg}}_{{\rm{j}}})-\,{\rm{\min }}({{\rm{Rg}}}_{1}{:\mathrm{Rg}}_{{\rm{j}}})],$$

with Rg_i_ being the range of species *i* and max(Rg_1_:Rg_j_) and min(Rg_1_:Rg_j_) being the maximum and minimum values of the ranges in the set of *j* species, respectively. Additionally, for each species and each climatic variable, the average of all observed values across all the grid cells of the species geographic range was calculated. These mean values were used to define the species’ niche position. The niche width and niche position of each family were computed following the same protocol as above, considering the distribution area occupied by all the species in the family as the family’s distribution range.

We also explored an alternative approach, computing family niche width after summarising the six climatic variables through a principal components analysis (PCA). This yielded equivalent results. Specifically, the first two PCA axes accounted for 97% of the variance among the climatic variables, and family niche widths based on these two PCA axes were highly correlated (Pearson’s *r* = 0.937) with niche widths computed as described above (Appendix S12).

The mean species niche width for a family was computed as the average value of all the species’ climatic niche widths in that family. Finally, the geographic extent (in km^2^) of each family was also computed, again considering the union of the ranges of all the species in the family as the range. All GIS analyses were conducted in R using the packages *rgdal*^45^, *geosphere*^46^, *maptools*^47^, and *raster*^43^. Details on R functions are provided in the Supplementary Material (Appendix S1).

We acknowledge that outliers or erroneous localities could cause errors in our estimates of climatic niche width. However, this should be a source of random error, and not bias. Further, our use of range maps (rather than point localities) to estimate climate niche values should tend to ameliorate rather than exacerbate such effects.

### Diversification rates

The net diversification rate for each family was estimated given the species richness and age of each family. The species richness of each family was based on the number of species in each family for which climatic niche data were obtained. These data include all species included in the IUCN^40^ database (with exception of three species for which the niche could not be computed) and guarantees that both family species richness and niche width are computed for exactly the same set of species. Ages of families were estimated from a time-calibrated phylogeny of mammals^34^. The phylogeny is based on a concatenated analysis of 26 genes, and the divergence dates were estimated incorporating autocorrelated evolutionary rates and hard-bounded age constraints. Although other mammal phylogenies are available, this phylogeny is based on extensive molecular data and is well-resolved at the family level. This phylogeny includes 164 mammal species, representing 147 terrestrial and marine families. Four terrestrial families (i.e. Aotidae, Lepilemuridae, Pitheciidae, and Platacanthomyidae) were not included in this phylogeny and were therefore excluded here. However, these four families collectively include only 80 species, and so their exclusion should have limited impact on the results. Diversification rates could be computed for all the terrestrial families included in the tree.

Family diversification rates were estimated following the method-of-moments estimator for stem groups described in Magallón & Sanderson^48^ (their equation 6). This method typically incorporates an assumed value of the relative extinction rate (ε = extinction rate/speciation rate) for calculating diversification rates:

$${\hat{r}}_{\varepsilon }=\frac{1}{t}\,\mathrm{ln}[n(1-\varepsilon )+\varepsilon ]$$

${\hat{r}}_{\varepsilon }$ represents diversification rate under a certain relative extinction fraction (ε), which accounts for the fact that only clades that survive to the present day are included (and thus might lead to bias in estimating rates). *t* is the family stem group age (i.e. the time of divergence between a family and its sister clade, so that the stem group age of two sister families is the same). n is the number of extant species in the family. Following standard practice, three different values for the relative extinction fraction were assumed (ε = 0, no extinction; ε = 0.45, intermediate rate; and ε = 0.9, high rate). Results were similar using all three values. Therefore, for brevity, only the results for ε = 0.45 are presented in the main text (see Appendix S3 in Supplementary Material for analyses using ε = 0 and ε = 0.9). We used the stem-group estimator because it is more accurate in simulations than the crown-group estimator and (unlike the crown-group estimator) is not affected by incomplete taxon sampling within clades^49^.

Some authors have claimed that the net diversification rate estimator requires that diversification rates must be constant within clades^50,51^, and that they should therefore only be used if there is a positive relationship between clade age and richness among clades. However, these authors did not actually address the accuracy of this estimator. Simulations that did address its accuracy show that it yields strong relationships between true and estimated diversification rates, regardless of the relationship between clade ages and richness^52^ and regardless of whether rates are homogeneous or heterogeneous within clades^49^. Furthermore, this estimator will correctly reflect that young clades with many species have high net diversification rates (and older clades with fewer species have lower rates), regardless of variation in instantaneous diversification rates within clades over time. Simulations also show that diversification rates (speciation – extinction) can be informative for predicting richness patterns even when there are strong ecological limits on richness^53^. However, variation in diversification rates over time could potentially uncouple net diversification rates from clade richness^4,52^, for example, if fast rates in young clades fail to generate high richness due to declining diversification rates over time. Therefore, the relationship between diversification rate and species richness was assessed (see Appendix S5 in Supplementary Material) in order to test if the differences in diversification rates between families are relevant to explaining richness patterns^4^. We also conducted analyses using ln-richness instead of diversification rates as the dependent variable, as recommended by authors who argue that net diversification rates require constant rates within clades^14,50,51^.

We note that many possible approaches are available to analysing diversification. The approach that we use here focuses on estimating net rates for individual clades, which is our main focus. Therefore, the estimator that we used here should be the most appropriate for our research question. This is also the same estimator used in previous studies on this topic^12,15–17^, which allows our results to be directly compared to earlier studies.

### Rates of niche evolution

Rates of niche evolution were calculated separately for each family and for both temperature and precipitation. Rates were calculated based on the phylogeny of species within each family, using species values for annual mean temperature (BIO1) and annual mean precipitation (BIO12), using average values across the range of each species. We focused on these two variables because they should reflect the most important aspects of the species climatic distribution (e.g., BIO1: tropical vs. temperate; BIO12: arid vs. mesic), and more so than short-term, extreme values (BIO5, BIO6, BIO16, BIO17). For the phylogeny within each family, we used the species-level tree of mammals from Rolland *et al*.^11^ (their maximum clade credibility consensus tree). These authors generated this tree by redating the species-level tree of Bininda-Emonds *et al*.^33^ (as modified by Fritz *et al*.^54^) using dates from Meredith *et al*.^34^. They also randomly resolved polytomies in this tree using the method of Kuhn *et al*.^55^ to generate 100 trees, and then obtained a consensus tree from those 100 trees. We conducted analyses on this consensus tree. Only one terrestrial family (Diatomyidae) was not represented in this phylogeny. This tree includes 5020 terrestrial and marine mammal species.

The rate of niche evolution was estimated as the sigma parameter of a Brownian-motion model of evolution (for details see O’Meara *et al*.^56^). The lambda model of evolution^57^ actually showed a better fit both for BIO1 and BIO12 than the Brownian-motion model (or than the Ornstein-Uhlenbeck [OU] model, see Table S6.1 in Supplementary Material), but the sigma parameter of a lambda or a OU model could only be computed for families with three or more species in the tree (76 families), which would further reduce the dataset. However, sigma values calculated using the lambda and Brownian-motion models have a strong, positive correlation (Pearson’s *r* = 0.90 for BIO1 and *r* = 0.80 for BIO12, see Fig. S6.1 in Supplementary Material), as expected given the close relationship between these models when lambda is high (i.e. fitted lambda = 0.89 for both BIO1 and BIO12, and lambda = 1 is the Brownian-motion model). Therefore, the sigma values for the Brownian-motion model were used, as they could be computed for more families. Furthermore, using the same set of families in all phylogenetic regressions is necessary in order to partition the variance among competing models. The sigma parameter was computed for all monophyletic families with two or more species in the tree with the command *fitContinuous* in the R package *geiger*^58^. All families that were monospecific (*n* = 20) or paraphyletic (*n* = 17) in the tree of Rolland *et al*.^11^ were excluded, since estimation of ages, richness, and rates of niche evolution and diversification would be problematic for these families. We also excluded two families that each had two species in the IUCN database but with only one of those species in the tree (Mystacinidae and Myzopodidae). Therefore, in all subsequent analyses, the dataset was composed of 92 families (spanning 3335 species).

We also performed alternative analyses to address whether our estimates of rates of niche evolution were affected by the algorithms used to resolve polytomies^59^. We tested the correlation between our niche-evolution rates (from the tree of Rolland *et al*.^11^) and an alternative set of rates for 18 mammal families computed from alternative trees. These trees were from Arnold *et al*.^60^, and included version 3 of the primates dataset (based on sequences from 17 genes) and version 1 of the Perissodactyla (15 genes), Cetartiodactyla (20 genes), and Carnivora (29 genes) datasets (see Appendix S7 in Supplementary Material). The niche evolution rates computed for these families from these two sources were strongly correlated (Pearson’s *r = *0.91, *P* < 0.001 for temperature niche rate, and *r* = 0.70, *P* = 0.001 for precipitation niche rate). Therefore, we considered the estimates of niche evolution rate derived from the tree of Rolland *et al*.^11^ to be robust and used them in subsequent analyses. We also note that the strong phylogenetic signal in the temperature and precipitation variables (see above) suggests that random resolution of polytomies within families has not strongly influenced our inferences of evolutionary patterns in these variables (i.e. if random resolutions had a strong effect, there should be limited phylogenetic signal instead of lambda values close to 1). Furthermore, random resolution of polytomies should have no impact on our estimates of diversification rates.

### Phylogenetic regression

Regression analyses were conducted using phylogenetic methods to control for the effect of shared phylogenetic history on diversification rates and niche attributes among families^61^. We conducted phylogenetic generalized least-squares regressions^62^ (PGLS) using the R package *caper*^63^. We used lambda values estimated by maximum likelihood to transform branch lengths and with kappa and delta values both set to 1.

The relationship between family niche width and mean species niche width can indicate levels of niche divergence among species within a family^17^. A PGLS between family niche width and mean species niche width was fitted and the residuals of this relationship were used as a proxy for niche divergence. With perfect niche conservatism among the species in a family, mean species niche width and family niche width would be the same, with the residuals of the relationship close to zero. Therefore, residuals reflect non-overlap of species niches within families (i.e. niche divergence^17^). However, we acknowledge that this index may not be as informative as using direct estimates of rates of niche evolution, and comparing these indices is one of our goals here. For example, niche divergence ignores the phylogeny within clades, whereas rates of niche change incorporate this information.

Five linear regression analyses were conducted to evaluate the hypotheses presented in Gómez-Rodríguez *et al*.^17^ Specifically, we assessed the relationships between diversification rate and (i) family niche width, (ii) mean species niche width, (iii) family niche divergence, (iv) family niche position for each individual climatic variable (i.e. defined as the mean climatic conditions across the geographic range of the family), and (v) family geographic extent. Given that niche position was defined by six variables, a forward stepwise procedure was used to select the most parsimonious model (based on *F*-statistic) explaining diversification rate. We note that our index of family niche position is not weighted towards the climatic conditions where most species in a family occur. However, using an index that does include such a weighting (i.e. taking the mean of species means across species in each family) yields values of niche position that are strongly correlated with the index that we used (Pearson’s *r* between indices among families: BIO1 = 0.896, BIO5 = 0.875, BIO6 = 0.881, BIO12 = 0.830, BIO16 = 0.899, BIO17 = 0.785).

We also compared the explanatory power of niche divergence (the aforementioned residuals of family vs. mean species niche width) relative to the rate of niche evolution. First, a bivariate linear regression was conducted to assess the relationships between niche divergence and niche evolution rate for BIO1 (hereafter “rate of temperature niche evolution”) and niche evolution rate for BIO12 (hereafter “rate of precipitation niche evolution”). Second, two linear regressions were conducted to test the relationships between diversification rate and rate of temperature niche evolution and rate of precipitation niche evolution. Third, we performed a multiple regression analysis including both temperate and precipitation niche rates as independent variables (this should be more comparable to niche divergence, which combines both temperature and precipitation). Finally, to partition the unique contribution of those models explaining an important portion of the variance in diversification rate (i.e. ~20% or more), four models (i.e. family niche width, geographic extent, niche divergence, and niche evolution rate) were combined in a full model and variance partitioning was used to compute their unique and joint contributions to the explained variance.

We performed null models in order to assess if the observed relationships between niche width and diversification rate appear because families with more species span more divergent climatic conditions due to sampling alone (H0). We tested the relationship between (a) family niche width vs. mean species niche width, (b) diversification rate vs. family niche width, (c) diversification rate vs. mean species niche width, and (d) diversification rate vs. niche divergence (residuals of regression “a”) using PGLS in the null families. We sampled with replacement 92 null families with the same species richness as the original ones. Three different null models were considered: (1) species sampled from the pool of all mammal species (unconstrained model), (2) species sampled from the pool of species within the latitudinal and longitudinal range of the original family (spatially constrained model), and (3) species sampled from the pool of species within the original family climatic niche (climatically constrained model). We took 1000 samples of 92 null families and compared the distribution of the *r*^2^ of the aforementioned PGLS among these 1000 replicates with the observed *r*^2^ in the original families.

An additional robustness test was performed to ensure that results were not influenced by the different ages of clades. We defined major clades of similar age in the species-level tree of Bininda-Emonds *et al*.^33^ with dates from Meredith *et al*.^34^, regardless of whether they corresponded to a named higher taxon or not. Specifically, we went through the tree and identified the clades with stem ages that were closest to the average stem age of all families (41.65 Ma), but with the restriction that the clades selected were older than that mean age. We then computed climatic niches and niche evolution rates for these clades, excluding those that were monospecific (86 clades were used in these analyses). We conducted the same regression analyses and variance partitioning described before, with the richness of these clades as the dependent variable.

All variables (unless otherwise noted in the Results) were logarithmically transformed (natural logarithm) to improve the normality of model residuals. All analyses were conducted in R. Raw data (e.g., diversification rates, niche widths, niche evolution rates) used for statistical analyses are provided in the Supplementary Material (Appendix S2).

### Data Availability

Range maps of mammals are available at http://www.iucnredlist.org/technical-documents/spatial-data. Two published mammal phylogenies were used (species-level phylogeny in Rolland *et al*.^11^ and family-level phylogeny in Meredith *et al*.^34^). Climatic data were obtained from http://worldclim.org/current (Hijmans *et al*.^42^).

## Results

**Figure 1:**
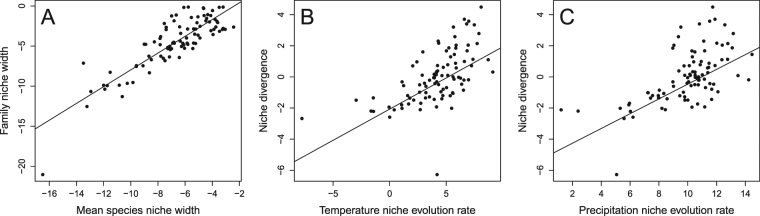
Scatterplots of the relationship between family niche width and mean species niche width (**A**) and between niche divergence and rate of temperature niche evolution (**B**) and rate of precipitation niche evolution (**C**). Note that the residuals of the model represented in Figure A correspond to the variable defined as “niche divergence”. Phylogenetic generalized least-squares models are superimposed. All variables (except niche divergence) are ln-transformed.

**Figure 2:**
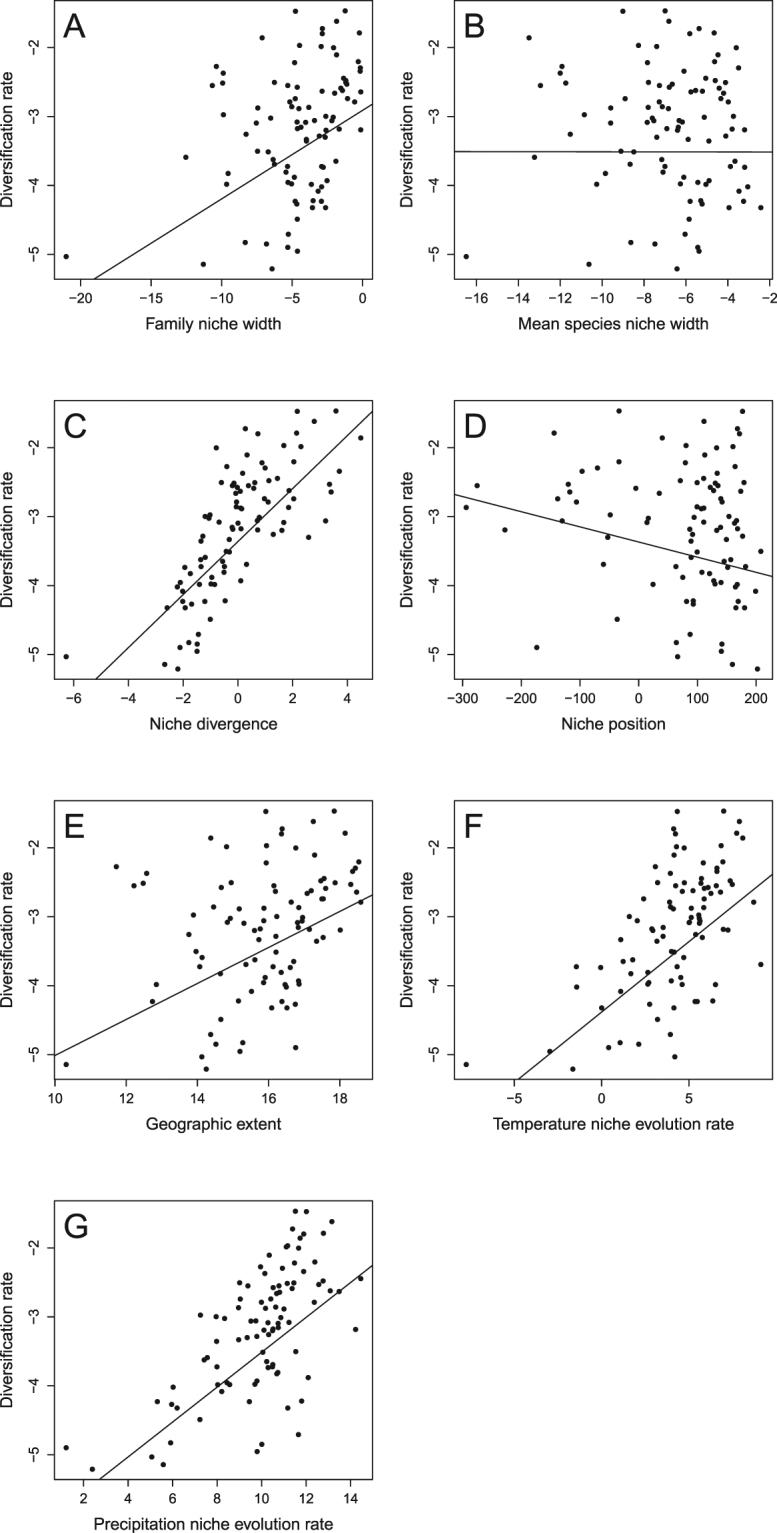
Scatterplots showing the relationships between diversification rate and family niche width (**A**), mean species niche width (**B**), niche divergence (**C**), niche position (**D**), geographic extent (**E**), rate of temperature niche evolution (**F**) and rate of precipitation niche evolution (**G**). Phylogenetic generalized least-squares regression lines are superimposed. All variables (except niche divergence and niche position) are ln-transformed.

A positive and strong relationship was observed between family niche width and mean species niche width (*r*^2^ = 0.73, *F*_1,90_ = 243.8, *P* < 0.001, *n* = 92 families, Fig. 1A). The relationship between diversification rate and family niche width was positive and significant (*r*^2^ = 0.20, *F*_1,90_ = 22.92, *P* < 0.001, Table 1, Fig. 2A). In contrast, there was not a significant relationship between diversification rate and mean species niche width (*r*^2^ < 0.001, *F*_1,90_ < 0.001, *P* = 0.99; Table 1, Fig. 2B). A strong positive relationship (*r*^2^ = 0.59, *F*_1,90_ = 130, *P* < 0.001; Table 1, Fig. 2C) was observed between diversification rate and absolute niche divergence (i.e. the residuals of the family niche width vs. mean species niche width relationship). A significant, positive relationship was also observed between diversification rate and geographic extent (*r*^2^ = 0.19, *F*_1,90_ = 21.65, *P* < 0.001; Table 1, Fig. 2E) and a weak negative one with niche position (*r*^2^ = 0.07, *F*_1,90_ = 6.341, *P* = 0.014; Table 1, Fig. 2D). Niche position was defined by the minimum temperature of the coldest month (BIO6) after a forward stepwise procedure to select the most parsimonious model (see Appendix S8 in Supplementary Material). However, both geographic range area and niche position explained little variation in diversification rates relative to climatic niche divergence. There was a strong positive relationship between diversification rate and species richness of families (*r*^2^ = 0.79; *F*_1,90_ = 342.7, *P* < 0.001).

**Table 1:** Results from univariate phylogenetic generalized least-squares models testing the relationship between diversification rate (ε = 0.45) and different attributes of the family niche as well as with the geographic extent of the family.

	*r* ^2^	*F*	*P*	Slope (95% CI)
Family niche width	0.20	22.92	**<0**.**001**	0.129 (±0.053)
Mean species niche width	<0.001	<0.001	0.99	−0.000 (±0.075)
Niche divergence*	0.59	130	**<0**.**001**	0.384 (±0.066)
Niche position*	0.07	6.34	**0**.**014**	−0.002 (±0.002)
Geographic extent	0.19	21.65	**<0**.**001**	0.261 (±0.110)
Rate of temperature niche evolution	0.38	55.04	**<0**.**001**	0.204 (±0.054)
Rate of precipitation niche evolution	0.44	69.31	**<0**.**001**	0.253 (±0.060)

Significant *P*-values are marked in bold. F-values for 1 and 90 degrees of freedom, and the slopes of the relationships with a 95% confidence interval are also provided.

^*^Variable not ln-transformed.

Niche divergence showed a positive relationship with rates of temperature and precipitation niche evolution in a bivariate model (*r*^2^ = 0.50, *F*_2, 89_ = 44.87, *P* < 0.001). This shows that greater niche divergence among species within a family is generally related to a higher rate of niche evolution in that family. A strong positive relationship was also found between diversification rate and rate of temperature-niche evolution (*r*^2^ = 0.38, *F*_1,90_ = 55.04, *P* < 0.001; Table 1, Fig. 2F) and rate of precipitation-niche evolution (*r*^2^ = 0.44, *F*_1,90_ = 69.31, *P* < 0.001; Table 1, Fig. 2G). A multiple regression model including rates of both temperature and precipitation niche evolution explained more than half of the variation in diversification rates (*r*^2^ = 0.51, *F*_1,89_ = 46.73, *P* < 0.001), similar to the level explained by niche divergence.

**Figure 3:**
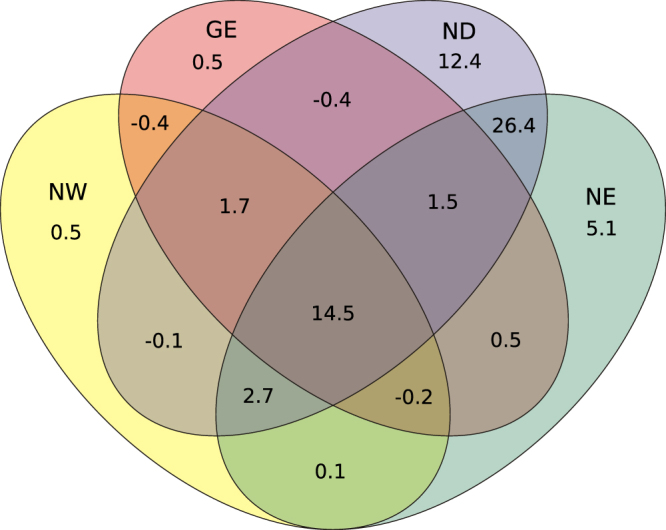
Venn diagram showing the results of variance partitioning on a full model of diversification rate with family niche width (NW), geographic extent (GE), niche divergence (ND), and niche evolution rate (NE) as explanatory variables. Results are shown as percentage of explained variance.

A model assessing the relationship between diversification rate and all independent variables explaining an important portion of its variance was also tested. These independent variables included family niche width, geographic extent, rate of niche evolution (with temperature and precipitation rates as independent variables), and niche divergence from the residuals of the regression of family niche width vs. mean species niche width. This model explained a large proportion of the variation in diversification rates (*r*^2^ = 0.65, *F*5,86 = 31.55, *P* < 0.001). Variance partitioning (Fig. 3) showed that most of the variance explained by this model (64.7% explained variance) was shared among the variables (46.3%). The variance explained exclusively by family niche width (0.5%) and geographic extent (0.5%) was negligible, and relatively low for rates of niche evolution (5.1%). Niche divergence was the variable with the largest unique contribution (12.4%). Most of the variance was shared between niche divergence and niche evolution rate (26.4%) and between all variables (14.5%).

To assess the robustness of our results regarding the estimates of diversification rates, the full model was conducted with species richness instead of diversification rate as the dependent variable, and results were similar. Family richness was strongly and positively related to niche divergence (*r*^2^ = 0.70, *F*_1,90_ = 207.9, *P* < 0.001), rate of temperature niche evolution (*r*^2^ = 0.34, *F*_1,90_ = 45.47, *P* < 0.001) and precipitation niche evolution rate (*r*^2^ = 0.38, *F*_1,90_ = 55.43, *P* < 0.001). In a variance partitioning of a full model, niche divergence was again the variable with the largest unique contribution (21.9%). Most of the variance was shared between niche divergence and rates of niche evolution (25.4%; see Appendix S4 in Supplementary Material).

In the null models (unconstrained, geographically constrained, and climatically constrained) in which we sampled 1000 replicates of 92 null families, the relationship between diversification rate and mean species niche width and between diversification rate and family niche width was generally stronger than the observed relationship (see Appendix S11). However, the relationship between diversification rate and niche divergence was comparable to the observed relationship only in the case of the unconstrained model (*P* = 0.056), and was weaker in the spatially and climatically constrained models (*P* <= 0.002 in both cases). The *r*^2^ in the relationship between family niche width and mean species niche width was higher in the null models (*P* <= 0.011 in the spatially and climatically constrained models), except in the unconstrained null model (*P* = 0.428). These results suggest that the observed relationship between diversification rate and niche divergence between species within families is comparable to sampling these species from the pool of all mammals, from any biome or world region.

We then used major clades stemming from the nodes closest to (but older than) the average family age (41.65 Ma) instead of families as units of analysis. Using these clades (mean stem age = 54.7 Ma, standard deviation = 20.2, *n* = 86), strong positive relationships were again found between clade richness and niche divergence (*r*^2^ = 0.58, *F*_1,84_ = 114.9, *P* < 0.001), rate of temperature-niche evolution (*r*^2^ = 0.46, *F*_1,84_ = 70.47, *P* < 0.001) and rate of precipitation-niche evolution (*r*^2^ = 0.46, *F*_1,84_ = 72.79, *P* < 0.001). Partitioning of variance in species richness among all predictors also yielded results similar to those reported above (Appendix S9 in Supplementary Material).

## Discussion

Overall, our results show that two measures of niche change among species within clades (absolute climatic niche divergence and rates of climatic niche evolution) are each strongly related to diversification rates among mammal clades. In regression models, absolute climatic niche divergence and rates of climatic niche evolution each explain over half of the variation in diversification rates among mammal families. Diversification rates in turn explain most (79%) of the variation in species richness. These results parallel those for amphibians^16,17^ and birds^15^, and together they suggest that changes in climatic niches are broadly important in understanding large-scale patterns of diversification and richness. We also show here that our measures of absolute niche divergence and climatic niche rates are strongly related, and explain similar amounts of variation in diversification rates.

These results are consistent with the hypothesis that speciation by climatic niche divergence is common and important^64,65^ (H2). Support was not found for the hypothesis that most speciation is linked to climatic niche conservatism (H1), since that hypothesis predicts a negative relationship between diversification rate and family-level niche width. The hypothesis that families with broader species-level niche widths have lower extinction rates (leading to higher diversification rates, H3) was also not supported, as this hypothesis predicts a strong relationship between diversification rate and mean species niche width^17^. We found that families containing species with wider niches do not tend to have higher diversification rates (and diversification rates reflect both speciation and extinction). Likewise, the unique contribution of geographic extent was negligible. Thus, even though the variation in diversification rate explained by family niche width and geographic extent was very similar, there was no strong support for geographic extent influencing diversification rates (H4). Surprisingly, only weak support was found for the hypothesis linking diversification rate and climatic regime (e.g., tropical vs. temperate; H5). This lack of a clear relationship is consistent with some previous studies in mammals^31,32^, but not others^11^. These discrepancies may be due to the different taxonomic levels at which analyses were performed. For example, our results include most mammal families and disagree with those from mammalian orders^11^, but that study only included the eight most species-rich orders, and did not include direct data on climate. We also did not find any evidence of narrower species-level niche widths being associated with higher diversification rates^30^. In this case, the discrepancies with previous studies may be due to methodological differences. Specifically, our measure of niche width was continuous and not a binary (i.e. specialist/generalist) and we analysed data at the level of families and clades, and not at the species level (as in Rolland & Salamin^30^). Furthermore, this previous study^30^ did not address the potential impact of narrower temperate niches in the tropics^23,24^ on their results (i.e. their results may reflect the impact of occurrence in the tropics on diversification, not niche widths directly: see H5 above). Overall, given these results, we favour the hypothesis that large-scale patterns of mammalian diversification are strongly influenced by a relationship between climatic niche change and speciation. The role of climatic niche divergence in mammalian speciation could be further explored by more detailed analyses within these families, such as looking for non-overlap in climatic niche models of sister species^65^. Furthermore, the relative roles of speciation and extinction in driving these overall patterns of diversification could be explored with additional clade-level analyses (e.g., analysing clades with well-sampled, species-level phylogenies using methods that estimate separate speciation and extinction rates).

Based on our results, we speculate that it is most likely that climatic niche change drives diversification rather than vice versa. It is very difficult to devise a plausible mechanism whereby diversification drives niche change that is not ruled out by our results. Clades could speciate with little change in their climatic niches, through niche conservatism, divergence on non-climatic niche axes, and/or non-ecological speciation processes. However, such processes should not generate a strong relationship between diversification rate and niche change. In theory, higher species richness of clades could lead to greater climatic niche divergence through geographic spread alone. Thus, the more diversification, the more divergence in climatic niches. But again, this should not increase the rate of climatic niche change (i.e. if niche divergence is a passive byproduct of speciation and range expansion). Furthermore, this scenario should generate a strong relationship between diversification rate, niche divergence, and clade range extent (area). Our results do not support this idea, as niche divergence has a strong relationship with diversification rate that is independent of clade area. In addition, the fact that the relationship between diversification rate and niche divergence is significantly stronger than would be expected under null assumptions (see Appendix S11) suggests that there are other mechanisms that link niche divergence and diversification rate other than families with more species having more divergent climatic niches due to sampling alone.

Our study includes two similar measures of climatic niche change within families (absolute climatic niche divergence and climatic niche evolution rates). These measures are significantly related, and explain similar amounts of variation in diversification rates. However, most previous studies have focused on niche evolution rate rather than absolute niche divergence. The positive relationship between diversification rates and rates of climatic niche evolution has been found in other groups (e.g., in plethodontid salamanders^12^, in the plant genus *Babiana* in the Cape flora^13^, in birds^15^, and in frogs^16^). These results suggest that families with faster rates of climatic niche evolution might diversify faster due to niche divergence promoting speciation or climatic niche lability buffering families from extinction (e.g., due to climatic fluctuations^12^). As mentioned above, we favour the hypothesis that niche divergence drives speciation, but this should be verified with additional species-level and clade-level analyses (e.g., estimating speciation and extinction rates separately).

Interestingly, in mammal families, faster rates of climatic niche evolution are not associated with species having narrower niche widths, as there is no relationship between rates of climatic niche evolution and mean species niche widths (see Appendix S10 in Supplementary Material). Other studies have found weak or positive relationships between niche width and rates of niche evolution (e.g., in salamanders^66^). They have also found faster rates of temperature niche evolution in temperate species, which typically have wider temperature niche widths (e.g., in mammals^27^, birds^26^, and across plants and animals^67^).

Our results showing the importance of climatic niche divergence for mammalian diversification might be seen as conflicting with previous studies showing broad-scale climatic niche conservatism in mammals^27,68^. For example, Buckley *et al*.^68^ suggested that global patterns of species richness in mammals were explained by phylogenetic conservatism in climatic niches. However, their study did not analyse diversification rates among clades. Furthermore, our results do show strong phylogenetic conservatism in climatic variables across all sampled mammal species (lambda = 0.89 for both BIO1 and BIO12). Most importantly, our results suggest that clades with higher diversification rates have faster rates of climatic niche divergence: this means that some clades show conservatism whereas others show rapid climatic niche divergence (and the latter diversify more quickly). Similarly, studies in plethodontid salamanders have also found that climatic niche conservatism drives spatial richness patterns^69,70^ whereas climatic niche divergence drives differences in diversification rates among clades, with stronger conservatism in clades that diversify more slowly^12^.

There are several potential sources of error in our study, but none that should overturn our major conclusions. First, climatic niches were estimated using geographic data from species range maps. Range maps are based on interpolations of locality data, and some maps might span grid cells outside the actual species range. On the other hand, use of range maps samples niche variables equally from of all parts of the species range, whereas estimating mean niche variables from locality data alone might over-represent some parts of the range relative to others. Thus, the use of range maps should be less problematic. Second, the climatic variables were measured at a slightly larger spatial grain than in some previous studies (~4.5 km vs. 1 km). This larger grain size might contribute some random error to the climatic data, especially for species that occur in montane tropical regions where climate varies extensively over small spatial scales. However, it seems very unlikely that these errors could introduce a systematic bias that explains the strong relationship between diversification rate and climatic niche divergence reported here. Third, the phylogeny and divergence dates within and between mammalian families may not be fully accurate, leading to potential errors in estimating diversification rates and rates of niche evolution. But again, it is unclear how random errors in clade ages could generate a significant non-random relationship between diversification rates and climatic niche variables. Similarly, even if all the estimated ages were non-randomly biased (e.g., older than the actual ages), this would not necessarily overturn our conclusions (i.e. younger clades with high richness would still have high diversification rates). Furthermore, we used alternative measures of diversification (richness) and niche change that do not depend on the phylogeny or clade ages, and found that they gave similar results to the phylogeny-based measures. Fourth, some authors have claimed that the use of these net diversification rate estimators requires that rates of diversification are constant within clades^50^. However, the stem-age estimators require only clade age and richness (and yield the same net rate regardless of whether rates are constant or variable within clades over time or among subclades), and the overall accuracy of these estimators is supported by recent simulations^52^ including ones that incorporate heterogeneous rates within clades^49^. We also show that diversification rates are strongly linked to richness patterns across mammalian families, and that we obtain similar results using richness instead of diversification rates.

In summary, our results here shed light on patterns of variation in diversification rates in one of the major clades of vertebrates. We find that two related measures of niche change within families (rates of climatic niche evolution and climatic niche divergence) each explain >50% of the variation in diversification rates among mammalian families. These results suggest that climatic niche divergence may be broadly important for speciation in mammals, given that our results are less consistent with alternative hypotheses to explain this relationship. More broadly, our results are similar to those from parallel analyses in plants^13^, amphibians^12,16,17^, and birds^15^. The similarity in results is particularly intriguing given the very different biologies of plants and animals and the very different physiologies and ecologies of amphibians and mammals (i.e. predominantly terrestrial endotherms vs. ectotherms that are typically at least partially aquatic^71^). Given these results, we speculate that similar processes might explain patterns of diversification and species richness in many other major clades across the Tree of Life.

## Electronic supplementary material

Supplementary Material

## References

[1] Futuyma, D. J. *Evolution*. (Sinauer Associates, 2013).

[2] Nee S, Mooers AO, Harvey PH (1992). Tempo and mode of evolution revealed from molecular phylogenies. Proc. Natl. Acad. Sci. USA.

[3] Ricklefs RE (2007). Estimating diversification rates from phylogenetic information. Trends Ecol. Evol..

[4] Wiens JJ (2011). The causes of species richness patterns across space, time, and clades and the role of “ecological limits”. Q. Rev. Biol..

[5] Scholl JP, Wiens JJ (2016). Diversification rates and species richness across the Tree of Life. Proc. R. Soc. B Biol. Sci..

[6] Hutchinson GE (1957). Concluding remarks. *Cold Spring Harb*. Symp.

[7] Soberón J (2007). Grinnellian and Eltonian niches and geographic distributions of species. Ecol. Lett..

[8] Holt RD (2009). Bringing the Hutchinsonian niche into the 21st century: Ecological and evolutionary perspectives. Proc. Natl. Acad. Sci..

[9] Moritz C, Patton JL, Schneider CJ, Smith TB (2000). Diversification of rainforest faunas: an integrated molecular approach. Annu. Rev. Ecol. Syst..

[10] Hua X, Wiens JJ (2013). How does climate influence speciation?. Am. Nat..

[11] Rolland J, Condamine FL, Jiguet F, Morlon H (2014). Faster speciation and reduced extinction in the tropics contribute to the mammalian latitudinal diversity gradient. PLoS Biol..

[12] Kozak KH, Wiens JJ (2010). Accelerated rates of climatic-niche evolution underlie rapid species diversification. Ecol. Lett..

[13] Schnitzler J, Graham CH, Dormann CF, Schiffers K, Linder HP (2012). Climatic niche evolution and species diversification in the Cape flora, South Africa. J. Biogeogr..

[14] Title PO, Burns KJ (2015). Rates of climatic niche evolution are correlated with species richness in a large and ecologically diverse radiation of songbirds. Ecol. Lett..

[15] Cooney CR, Seddon N, Tobias JA (2016). Widespread correlations between climatic niche evolution and species diversification in birds. J. Anim. Ecol..

[16] Moen DS, Wiens JJ (2017). Microhabitat and climatic niche change explain patterns of diversification among frog families. Am. Nat..

[17] Gómez-Rodríguez C, Baselga A, Wiens JJ (2015). Is diversification rate related to climatic niche width?. Glob. Ecol. Biogeogr..

[18] Wiens JJ (2004). What is speciation and how should we study it?. Am. Nat..

[19] Rosenzweig, M. L. *Species Diversity in Space and Time*. (Cambridge University Press, 1995).

[20] Losos JB, Schluter D (2000). Analysis of an evolutionary species–area relationship. Nature.

[21] Cardillo M, Huxtable JS, Bromham L (2003). Geographic range size, life history and rates of diversification in Australian mammals. J. Evol. Biol..

[22] Mittelbach GG (2007). Evolution and the latitudinal diversity gradient: speciation, extinction and biogeography. Ecol. Lett..

[23] Ghalambor CK, Huey RB, Martin PR, Tewksbury JJ, Wang G (2006). Are mountain passes higher in the tropics? Janzen’s hypothesis revisited. Integr. Comp. Biol..

[24] Quintero I, Wiens JJ (2013). What determines the climatic niche width of species? The role of spatial and temporal climatic variation in three vertebrate clades. Glob. Ecol. Biogeogr..

[25] Pyron RA, Wiens JJ (2013). Large-scale phylogenetic analyses reveal the causes of high tropical amphibian diversity. Proc. R. Soc. B Biol. Sci..

[26] Lawson AM, Weir JT (2014). Latitudinal gradients in climatic-niche evolution accelerate trait evolution at high latitudes. Ecol. Lett..

[27] Cooper N, Freckleton RP, Jetz W (2011). Phylogenetic conservatism of environmental niches in mammals. Proc. R. Soc. B Biol. Sci..

[28] Cadena CD (2012). Latitude, elevational climatic zonation and speciation in New World vertebrates. Proc. R. Soc. B Biol. Sci..

[29] Price SA, Hopkins SSB, Smith KK, Roth VL (2012). Tempo of trophic evolution and its impact on mammalian diversification. Proc. Natl. Acad. Sci..

[30] Rolland J, Salamin N (2016). Niche width impacts vertebrate diversification. Glob. Ecol. Biogeogr..

[31] Soria-Carrasco V, Castresana J (2012). Diversification rates and the latitudinal gradient of diversity in mammals. Proc. R. Soc. B Biol. Sci..

[32] Weir JT, Schluter D (2007). The latitudinal gradient in recent speciation and extinction rates of birds and mammals. Science.

[33] Bininda-Emonds ORP (2007). The delayed rise of present-day mammals. Nature.

[34] Meredith RW (2011). Impacts of the Cretaceous terrestrial revolution and KPg extinction on mammal diversification. Science.

[35] Stadler T (2011). Mammalian phylogeny reveals recent diversification rate shifts. Proc. Natl. Acad. Sci. USA.

[36] Fabre P-H, Hautier L, Dimitrov D, Douzery EJP (2012). A glimpse on the pattern of rodent diversification: a phylogenetic approach. BMC Evol. Biol..

[37] Schenk JJ, Rowe KC, Steppan SJ (2013). Ecological opportunity and incumbency in the diversification of repeated continental colonizations by muroid rodents. Syst. Biol..

[38] Jansa SA, Barker FK, Voss RS (2014). The early diversification history of didelphid marsupials: a window into South America’s “splendid isolation”. Evolution.

[39] Shi JJ, Rabosky DL (2015). Speciation dynamics during the global radiation of extant bats. Evolution.

[40] IUCN. IUCN Red List of Threatened Species. Version 2013.2. (2013). Available at: http://www.iucnredlist.org/technical-documents/spatial-data. Accessed November 2013.

[41] Peterson, A. T. *et al*. *Ecological Niches and Geographic Distributions*. (Princeton University Press, 2011).

[42] Hijmans RJ, Cameron SE, Parra JL, Jones PG, Jarvis A (2005). Very high resolution interpolated climate surfaces for global land areas. Int. J. Climatol..

[43] Hijmans, R. J. raster: Geographic Data Analysis and Modeling. (2014).

[44] R Core Team. *R: A Language and Environment for Statistical Computing*. (R Foundation for Statistical Computing, 2016).

[45] Bivand, R., Keitt, T. & Rowlingson, B. rgdal: Bindings for the Geospatial Data Abstraction Library. (2014).

[46] Hijmans, R. J. geosphere: Spherical Trigonometry. (2014).

[47] Bivand, R. & Lewin-Koh, N. maptools: Tools for Reading and Handling Spatial Objects. https://CRAN.R-project.org/package=maptools. (2014).

[48] Magallón S, Sanderson MJ (2001). Absolute diversification rates in angiosperm clades. Evolution.

[49] Meyer ALS, Wiens JJ (2018). Estimating diversification rates for higher taxa: BAMM can give problematic estimate of rates and rate shifts. Evolution.

[50] Rabosky DL (2009). Ecological limits and diversification rate: alternative paradigms to explain the variation in species richness among clades and regions. Ecol. Lett..

[51] Rabosky DL, Adams DC (2012). Rates of morphological evolution are correlated with species richness in salamanders. Evolution.

[52] Kozak KH, Wiens JJ (2016). Testing the relationships between diversification, species richness, and trait evolution. Syst. Biol..

[53] Pontarp M, Wiens JJ (2017). The origin of species richness patterns along environmental gradients: uniting explanations based on time, diversification rate and carrying capacity. J. Biogeogr..

[54] Fritz SA, Bininda-Emonds ORP, Purvis A (2009). Geographical variation in predictors of mammalian extinction risk: big is bad, but only in the tropics. Ecol. Lett..

[55] Kuhn TS, Mooers AØ, Thomas GH (2011). A simple polytomy resolver for dated phylogenies. Methods Ecol. Evol..

[56] O’Meara BC, Ané C, Sanderson MJ, Wainwright PC (2006). Testing for different rates of continuous trait evolution using likelihood. Evolution.

[57] Pagel M (1999). Inferring the historical patterns of biological evolution. Nature.

[58] Harmon LJ, Weir JT, Brock CD, Glor RE, Challenger W (2008). GEIGER: investigating evolutionary radiations. Bioinformatics.

[59] Rabosky DL (2015). No substitute for real data: A cautionary note on the use of phylogenies from birth-death polytomy resolvers for downstream comparative analyses. Evolution.

[60] Arnold C, Matthews LJ, Nunn CL (2010). The 10kTrees website: A new online resource for primate phylogeny. Evol. Anthropol. Issues News Rev..

[61] Grafen, A. The phylogenetic regression. *Philos*. *Trans*. *R*. *Soc*. *Lond*. *B*. *Biol*. *Sci*. 119–157 (1989). 10.1098/rstb.1989.0106 2575770

[62] Freckleton RP, Harvey PH, Pagel M (2002). Phylogenetic analysis and comparative data: a test and review of evidence. Am. Nat..

[63] Orme, D. *et al*. caper: comparative analyses of phylogenetics and evolution in R. (2013).

[64] Kozak KH, Wiens JJ (2007). Climatic zonation drives latitudinal variation in speciation mechanisms. Proc. R. Soc. B Biol. Sci..

[65] Hua X, Wiens JJ (2010). Latitudinal variation in speciation mechanisms in frogs. Evolution.

[66] Fisher-Reid MC, Kozak KH, Wiens JJ (2012). How is the rate of climatic-niche evolution related to climatic-niche breadth?. Evolution.

[67] Jezkova T, Wiens JJ (2016). Rates of change in climatic niches in plant and animal populations are much slower than projected climate change. Proc. R. Soc. B Biol. Sci..

[68] Buckley LB (2010). 2010. Phylogeny, niche conservatism, and the latitudinal diversity gradient in mammals. Proc. R. Soc. B Biol. Sci..

[69] Kozak KH, Wiens JJ (2010). Niche conservatism drives elevational diversity patterns in Appalachian salamanders. American Naturalist.

[70] Kozak KH, Wiens JJ (2012). Phylogeny, ecology, and the origins of climate-richness relationships. Ecology.

[71] Pough, F. H., Janis, C. M. & Heiser, J. B. *Vertebrate Life*. (Benjamin Cummings, 2009).

