# Supplementary material for: Climatic niche divergence drives patterns of diversification and richness among mammal families
Source: Sci Rep. 2018 Jun 8;8:8781. doi: 10.1038/s41598-018-27068-y (PMC5993713; doi:10.1038/s41598-018-27068-y)

Supplementary material

Climatic niche divergence drives patterns of diversification and richness among mammal families

Adrián Castro-Insua[1*], Carola Gómez-Rodríguez[1], John J. Wiens[2] and Andrés Baselga[1]

[1]*Departamento de Zoología, Facultad de Biología, Universidad de Santiago de Compostela, Rúa Lope Gómez de Marzoa, 15782, Santiago de Compostela, Spain*

[2]*Department of Ecology and Evolutionary Biology, University of Arizona, Tucson, AZ 85721-0088 U.S.A.*

* Corresponding author: adrian.castro@usc.es

Appendix S1. R functions used for GIS analyses

Species niche width and position

```
library(raster)
library(rgdal)
library(maptools)

# Reading raster with climatic data (done for six climatic
# variables described in main text, here presented only for BIO1)
bio1 <- raster("./bio1.bil")

# Reading polygons with species distribution
mamm <- readOGR("./All_MAMMALS_NOV2013/", "All_MAMMALS_NOV2013")

# For each species
union.poly <- unionSpatialPolygons(sp.polys, rep(1, length(sp.polys)))
# Extracting values of each climatic variable for the species distribution
# polygon (here presented only for BIO1)
bio1.ext <- extract(bio1, union.poly, small = TRUE)[[1]]
# Minimum, maximum, and mean values were obtained for each species
# and climatic variable
```

Family niche width and position

```
library(raster)

# For each family
# All the polygons of the species of each family are stored in the object
# "fam.polys"
# Convert all the polygons of a family to one raster representing its
# distribution range
fam.rast <- rasterize(fam.polys, bio1, field = 1)

# Extracting values of each climatic variable for the family distribution
# raster (here presented only for BIO1)
bio1.ext <- fam.rast[] * bio1[]
# Minimum, maximum, and mean values were obtained for each family
# and climatic variable
```

Family distributional area

```
library(geosphere)

# The custom function gdal_polygonize.R (a function by
# John Baumgartner, available at
# <https://johnbaumgartner.wordpress.com/2012/07/26/getting-rasters-into-shape-
# from-r/> [accessed 15 February 2014])
# was used in order to convert family rasters to a polygon

gdal_polygonizeR <- function(x, outshape=NULL, gdalformat = 'ESRI Shapefile',
                             pypath=NULL, readpoly=TRUE, quiet=TRUE) {
  if (isTRUE(readpoly)) require(rgdal)
  if (is.null(pypath)) {
    pypath <- Sys.which("gdal_polygonize")
  }
  if (!file.exists(pypath)) stop("Can't find gdal_polygonize.py on your system.")
  owd <- getwd()
  on.exit(setwd(owd))
  setwd(dirname(pypath))
  if (!is.null(outshape)) {
    outshape <- sub('\\.shp$', '', outshape)
    f.exists <- file.exists(paste(outshape, c('shp', 'shx', 'dbf'), sep='.'))
    if (any(f.exists))
      stop(sprintf('File already exists: %s',
                   toString(paste(outshape, c('shp', 'shx', 'dbf'),
```

```
                                  sep='.')[f.exists])), call.=FALSE)
  } else outshape <- tempfile()
  if (is(x, 'Raster')) {
    require(raster)
    writeRaster(x, {f <- tempfile(fileext='.asc')})
    rastpath <- normalizePath(f)
  } else if (is.character(x)) {
    rastpath <- normalizePath(x)
  } else stop('x must be a file path (character string), or a Raster object.')
  system2('python', args=(sprintf('"%1$s" "%2$s" -f "%3$s" "%4$s.shp"',
                                  pypath, rastpath, gdalformat, outshape)))
  if (isTRUE(readpoly)) {
    shp <- readOGR(dirname(outshape), layer = basename(outshape),
                   verbose=!quiet)
    return(shp)
  }
  return(NULL)
}

# For each family
fam.poly <- gdal_polygonizeR(fam.rast)

# Specify latlong projection to that polygon
fam.poly@proj4string <-
        CRS("+proj=longlat +datum=WGS84 +no_defs +ellps=WGS84 +towgs84=0,0,0")

# Area (in km^2) of the polygon calculated using the function areaPolygon()
# from package "geosphere"
area.km2 <- sum(areaPolygon(fam.poly)) / 1e+6
```

Appendix S2. Data for each mammal family, including stem group age (millions of years ago), species richness, diversification rate in events per million years (using a relative extinction fraction of ε = 0, ε = 0.45, and ε = 0.9), geographic extent, family niche width, mean species niche width, temperature (T) niche evolution rate (BIO1), and precipitation (P) niche evolution rate (BIO12). Niche widths are standardized and ln-transformed. Rates of niche evolution were only estimated for monophyletic families with more than one species in the phylogenetic tree used.

Family	Stem age	Species richness	Diversification rate			Extent (km^2)	Family niche width	Mean species niche width	T niche evolution rate	P niche evolution rate
			ε = 0.0	ε = 0.45	ε = 0.9					
**Acrobatidae**	48.5	2	0.0143	0.009	0.002	1744594	-5.27	-6.04	51.3	115325.7
**Anomaluridae**	54.7	7	0.0356	0.0267	0.0086	6036800	-6.35	-7.15	5.9	1666.3
**Atelidae**	15.5	28	0.2149	0.1782	0.0844	12994908	-2.86	-5.37	62	88550.7
**Bathyergidae**	38.4	15	0.0706	0.0564	0.0228	7789625	-7.44	-9.59	170.3	26279.5
**Bovidae**	18.7	134	0.2622	0.2305	0.1424	56071127	-1.24	-6.99	1086.5	100692.3
**Bradypodidae**	26	4	0.0533	0.0375	0.0101	10896101	-3.07	-4.1	33.8	17805.8
**Burramyidae**	48.2	5	0.0334	0.0241	0.007	1283808	-5.32	-7	75.4	35839.9
**Caenolestidae**	90.8	6	0.0197	0.0146	0.0045	340216	-4.78	-5.8	211	12722.1
**Callitrichidae**	13.8	42	0.2712	0.2293	0.1182	8211439	-4.76	-9.01	76.3	165060.5
**Calomyscidae**	30.9	8	0.0673	0.0511	0.0172	1075536	-9.87	-10.85	260.5	1409.2
**Camelidae**	66	3	0.0166	0.0112	0.0028	2323493	-4.63	-5.85	24.6	1377.3
**Canidae**	48.4	35	0.0734	0.0615	0.0306	118204955	-0.57	-3.98	6068.8	235383.9
**Capromyidae**	19.7	13	0.1302	0.1029	0.04	123282	-10.37	-11.91	21.7	21016.9
**Castoridae**	58.7	2	0.0118	0.0075	0.0016	18975764	-5.31	-5.43	1.5	3.4
**Cebidae**	13.8	17	0.2056	0.1656	0.0693	12758010	-2.86	-5.82	68.7	146403.1
**Cercopithecidae**	21.3	123	0.2261	0.1983	0.1212	31183190	-1.85	-6.82	2670.9	518810
**Cervidae**	20.3	54	0.196	0.1674	0.0905	76143249	-0.23	-4.65	2285.3	354795.5
**Cheirogaleidae**	29.9	29	0.1125	0.0934	0.0446	289204	-9.89	-12	51.3	25503.8
**Chinchillidae**	27.2	6	0.066	0.0487	0.0149	3029534	-6.52	-7.61	269.2	4175.6
**Chrysochloridae**	64.5	21	0.0472	0.0385	0.017	946762	-8.27	-11.52	218.7	30256.5
**Ctenodactylidae**	42.2	5	0.0382	0.0276	0.008	1371877	-12.54	-13.23	110.3	1919
**Ctenomyidae**	22.5	60	0.1819	0.1559	0.0858	1749335	-7.13	-13.49	3242.8	124491.7
**Cuniculidae**	30.1	2	0.023	0.0146	0.0032	12901337	-2.92	-3.25	239.2	203.2
**Cynocephalidae**	80.2	2	0.0086	0.0055	0.0012	1542056	-6.42	-6.43	0.2	10.9
**Dasyproctidae**	28.2	13	0.091	0.0719	0.028	10754521	-2.88	-5.22	101.7	719486.2
**Dasyuridae**	33.9	72	0.1261	0.1088	0.0617	8304427	-4.82	-7.84	337.8	96656.5
**Didelphidae**	81	98	0.0566	0.0493	0.0292	23085640	-2.09	-5.13	169.8	52269.2
**Dipodidae**	51.6	50	0.0758	0.0645	0.0344	39652782	-4.78	-8.9	355.1	8447.9
**Elephantidae**	62	2	0.0112	0.0071	0.0015	3975154	-4.62	-5.38	0.1	18015.9
**Emballonuridae**	52.2	52	0.0758	0.0646	0.0347	41156101	-1.07	-4.33	11.1	33245.4
**Equidae**	58.5	7	0.0333	0.0249	0.008	4695786	-6.26	-8.67	9117.6	36471.6
**Erinaceidae**	70.9	24	0.0448	0.0369	0.0168	41049823	-2.66	-7.39	319.4	11524.9
**Felidae**	29.8	36	0.1201	0.1008	0.0504	101564537	-0.16	-3.49	738.3	56164.1
**Furipteridae**	33	2	0.021	0.0133	0.0029	9686263	-3.56	-3.94	62.9	71473.9
**Galagidae**	37.9	18	0.0762	0.0616	0.0262	16749518	-5.16	-7.27	49.1	21857.6
**Giraffidae**	20.1	2	0.0345	0.0218	0.0047	2294613	-9.54	-9.84	5.3	43668.7
**Gliridae**	60.4	28	0.0552	0.0458	0.0217	19997589	-4.04	-7.85	150.4	29375.1
**Herpestidae**	24.2	34	0.1455	0.1218	0.0602	32336739	-1.85	-4.47	63.4	31076.4
**Hippopotamidae**	55.9	2	0.0124	0.0078	0.0017	2021067	-6.82	-7.48	8.4	22232.9
**Hipposideridae**	44.3	84	0.1	0.0867	0.0503	42164259	-1.35	-5.06	304.5	1902269.2
**Hominidae**	16.2	6	0.1107	0.0817	0.0251	3104826	-6.26	-7.79	24.7	8209.3

Family	Stem age	Species richness	Diversification rate			Extent (km^2)	Family niche width	Mean species niche width	T niche evolution rate	P niche evolution rate
			ε = 0.0	ε = 0.45	ε = 0.9					
**Hyaenidae**	27.9	4	0.0497	0.0349	0.0094	34164485	-3.99	-4.91	23.8	2916.3
**Hylobatidae**	16.2	16	0.1713	0.1375	0.0566	2725783	-2.94	-7.4	72.9	66876.2
**Hystricidae**	45.1	11	0.0532	0.0415	0.0154	27917086	-1.66	-3.8	1063.6	1507282.9
**Indriidae**	29.9	18	0.0965	0.0781	0.0332	202527	-10.66	-12.94	69.4	11907.2
**Lemuridae**	30.7	21	0.0991	0.0809	0.0358	262139	-9.93	-11.74	295.1	69740.4
**Leporidae**	49.8	62	0.0829	0.0712	0.0394	105215759	-0.13	-5.76	906.5	49595.8
**Lorisidae**	37.9	10	0.0607	0.047	0.0169	7749835	-3.41	-6.33	7.7	17017.7
**Macropodidae**	25.5	63	0.1626	0.1397	0.0775	8369975	-4.47	-8.26	939.8	71100.7
**Macroscelididae**	76.5	17	0.037	0.0298	0.0125	10935530	-6.7	-8.49	64	23295
**Manidae**	80.4	8	0.0259	0.0196	0.0066	20581970	-2.53	-4.9	54.1	17854.1
**Megadermatidae**	44.7	5	0.036	0.026	0.0075	17962591	-1.9	-3.66	3.4	27724.5
**Megalonychidae**	26	2	0.0267	0.0169	0.0037	5491962	-3.16	-3.47	3	3672.9
**Molossidae**	49.3	100	0.0935	0.0815	0.0485	57444294	-1.19	-4.11	114.3	93905.9
**Mormoopidae**	33.8	9	0.0651	0.0499	0.0174	11165117	-2.61	-3.74	4.9	2858.2
**Moschidae**	18.7	7	0.1042	0.0781	0.0252	10302535	-5.31	-7.27	710.4	48091.9
**Myrmecophagidae**	41.3	3	0.0266	0.018	0.0044	14606567	-2.91	-3.05	0.2	413.1
**Natalidae**	52.4	11	0.0458	0.0357	0.0132	6754595	-3.99	-5.9	3	7864.6
**Noctilionidae**	33	2	0.021	0.0133	0.0029	14859794	-2.63	-2.43	1	493.7
**Notoryctidae**	67.1	2	0.0103	0.0065	0.0014	1360427	-21.02	-16.49	66.2	158.2
**Nycteridae**	52.2	16	0.0532	0.0426	0.0176	20417544	-4.37	-6.36	34	46633.3
**Ochotonidae**	49.8	30	0.0683	0.0569	0.0273	20744722	-3.82	-7.8	346.9	7796.7
**Pedetidae**	54.7	2	0.0127	0.008	0.0017	4297716	-8.33	-8.63	2.9	369.2
**Peramelidae**	30.7	18	0.0942	0.0761	0.0324	2349572	-4.64	-6.8	444.1	37017.6
**Petauridae**	40.7	11	0.0589	0.046	0.017	2788752	-4.65	-6.17	152.9	75488.5
**Phalangeridae**	48.2	26	0.0676	0.0558	0.026	3890476	-4.52	-6.84	61	60272.1
**Phyllostomidae**	33.8	173	0.1526	0.1351	0.0859	19041332	-2.06	-3.6	113.2	116327.1
**Prionodontidae**	29.8	2	0.0232	0.0147	0.0032	3805072	-3.51	-5.29	581.4	133054.9
**Procaviidae**	62	5	0.0259	0.0187	0.0054	20879586	-5.04	-6.26	14.1	16314.9
**Procyonidae**	30	14	0.0879	0.0699	0.0277	26201302	-1.98	-4.2	523.3	43295.2
**Pseudocheiridae**	40.7	18	0.071	0.0574	0.0244	1894912	-5.03	-7.13	51.8	41528.5
**Pteropodidae**	63.5	183	0.082	0.0727	0.0465	29378339	-1.43	-5.54	187.1	482020.2
**Rhinocerotidae**	52.3	5	0.0307	0.0222	0.0064	12641510	-5.44	-7.09	14.1	45733.1
**Rhinolophidae**	44.3	74	0.0971	0.0839	0.0478	38478070	-1.19	-4.6	1575.6	349130
**Rhinopomatidae**	53	3	0.0207	0.014	0.0034	18805492	-4.67	-5.23	15.7	386.4
**Sciuridae**	52.4	279	0.1075	0.0961	0.0641	94058541	-0.18	-6.09	737.3	145891.1
**Solenodontidae**	75	2	0.0092	0.0058	0.0013	30151	-11.3	-10.63	0	265.2
**Soricidae**	67	375	0.0885	0.0796	0.0545	89142599	-1.12	-6.66	1774.7	288023
**Suidae**	31.1	18	0.0929	0.0751	0.0319	44088398	-1.55	-4.41	357.9	86927
**Tachyglossidae**	47.2	4	0.0294	0.0206	0.0056	8061611	-4.84	-6.1	93.1	178963
**Talpidae**	67	41	0.0555	0.0468	0.024	22767344	-2.18	-7.53	280.1	13638.8
**Tapiridae**	52.3	4	0.0265	0.0186	0.005	14096321	-3.64	-5.05	679.9	3066.1
**Tarsiidae**	59.3	10	0.0389	0.0301	0.0108	1157479	-7.45	-9.1	54.7	102327.5
**Tayassuidae**	31.1	3	0.0353	0.0239	0.0059	16305276	-2.79	-3.2	0.9	29505.6
**Tenrecidae**	64.5	33	0.0542	0.0453	0.0222	4439112	-7.54	-9.59	271	46328.2
**Thryonomyidae**	22.9	2	0.0303	0.0192	0.0042	7709923	-5.29	-5.43	14.9	4658.1
**Thyropteridae**	40.4	4	0.0343	0.0241	0.0065	10058317	-2.91	-3.88	0.2	2960.3
**Tragulidae**	42.7	10	0.0539	0.0418	0.015	7020197	-4.62	-6.91	17.8	36817.2

Family	Stem age	Species richness	Diversification rate			Extent (km^2)	Family niche width	Mean species niche width	T niche evolution rate	P niche evolution rate
			ε = 0.0	ε = 0.45	ε = 0.9					
**Tupaiidae**	57.3	18	0.0505	0.0408	0.0173	5973476	-2.6	-6.4	18.8	36091.9
**Ursidae**	38.4	8	0.0541	0.0411	0.0138	66363940	-0.14	-3.2	1388.9	24523.7
**Vespertilionidae**	49.3	417	0.1225	0.1104	0.0762	111856799	-0.31	-4.63	1035.6	241300
**Vombatidae**	39.8	3	0.0276	0.0186	0.0046	379792	-9.64	-10.26	100.7	5247.1

Appendix S3. Complementary analyses done for diversification rates estimated with relative extinction fractions of ε = 0.0 and ε = 0.9

Table S3.1. Results of the phylogenetic generalized least squares (PGLS) regression with diversification rate as the response variable

Results from PGLS models testing the relationship between diversification rate (ε = 0.0 and ε = 0.9) and the variable in the first column as the explanatory variable. Significant p-values are marked in bold. F-values for 1 and 90 degrees of freedom.

	**ε = 0.0**			**ε = 0.9**		
	r^2	F	P	r^2	F	P
**Family niche width**	0.21	23.4	**< 0.001**	0.20	23.05	**< 0.001**
**Mean sp. niche width**	< 0.01	0.003	0.96	< 0.01	0.11	0.74
**Niche divergence**	0.57	118.3	**< 0.001**	0.65	164.6	**< 0.001**
**Geographic extent**	0.19	21.37	**< 0.001**	0.21	23.58	**< 0.001**
**Niche position (BIO6)**	0.06	6.153	**0.015**	0.07	6.793	**0.011**
**Temperature niche evolution rate**	0.37	52.06	**< 0.001**	0.40	59.89	**< 0.001**
**Precipitation niche evolution rate**	0.42	65.32	**< 0.001**	0.45	74.41	**< 0.001**

Figure S3.1. Variance partitioning of the full model assessing the relationship between diversification rate (response variable) and family niche width (NW), geographic extent (GE), niche divergence (ND), and niche evolution rate (NE) as explanatory variables, with results for diversification rates calculated considering no extinction ($\varepsilon = 0$) and a high relative extinction fraction ($\varepsilon = 0.9$).

ε = 0.0

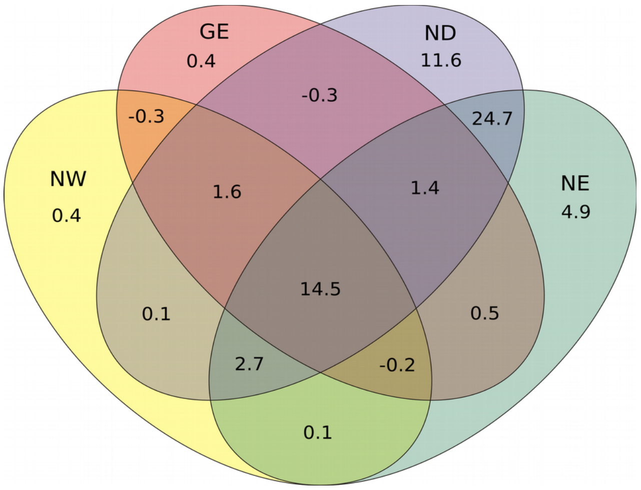

Full model: $r^2 = 0.62$, $F_{5, 86} = 28.16$, $p < 0.001$ (62.1% explained variance)

ε = 0.9

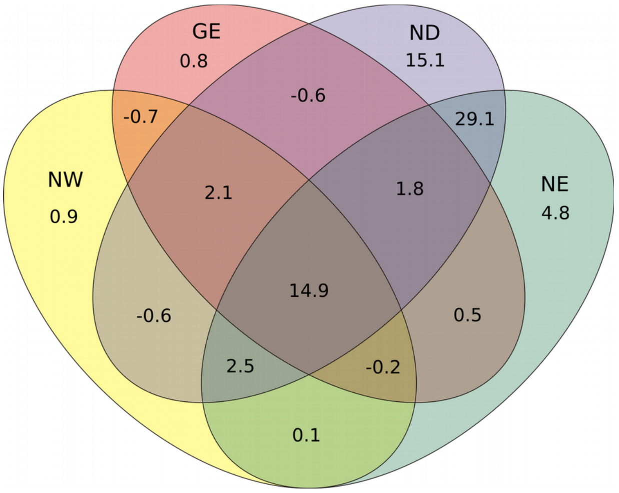

Full model: $r^2 = 0.71$, $F_{5, 86} = 41.32$, $p < 0.001$ (70.6% explained variance)

Appendix S4. Results of the variance partitioning on a full model of species richness (ln-transformed) as the dependent variable with family niche width (NW), geographic extent (GE), niche divergence (ND), and rate of niche evolution (NE) as explanatory variables. Results are shown as percentage of explained variance.

Full model: $r^2 = 0.76$, $F_{5, 86} = 54.18$, $P < 0.001$ (75.9% explained variance)

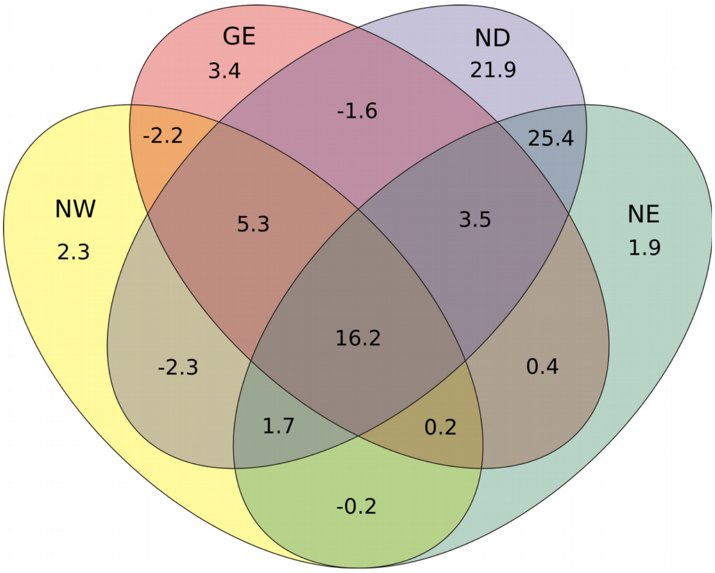

Appendix S5. Scatterplot of the relationship (r^2 = 0.79, $F_{1,90}$ = 342.7, P < 0.001) between diversification rate (ε = 0.45) and species richness (both variables were ln-transformed). The fitted PGLS model is superimposed.

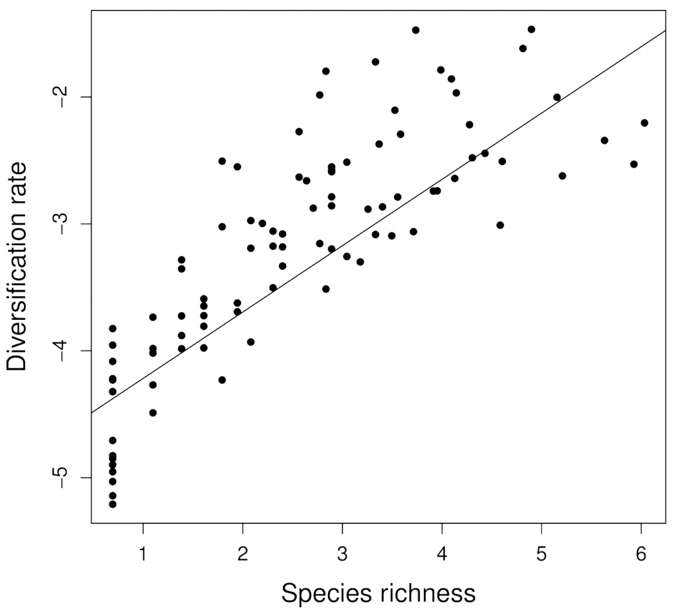

Appendix S6. Evolutionary models for the estimation of niche evolution rates.

Table S6.1. AIC values of the evolutionary models for rates of change in annual mean temperature (BIO1) and annual mean precipitation (BIO12). The lowest AIC (corresponding to the best-fitting model) is marked in bold.

	BM	OU	Lambda	White
Annual mean temperature (BIO1)	35315	33906	**32585**	34501
Annual mean precipitation (BIO12)	52004	49478	**47985**	49468

Figure S6.1. Scatterplots showing the relationship between the sigma parameter values computed using a Brownian-motion model of evolution (x-axis) and a lambda model (y-axis) for annual mean temperature (A) and annual mean precipitation (B). Both variables were ln-transformed. Pearson's *r* values measure the linear correlation between these estimates of niche evolution rate. Both estimates could be computed for 76 families.

A) Pearson's *r*: 0.90

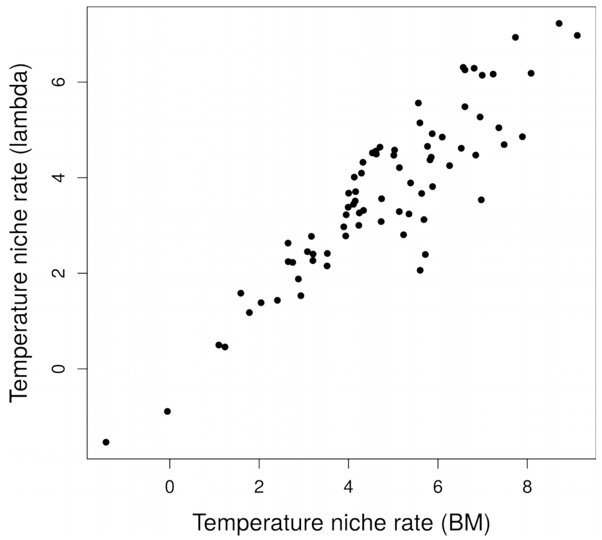

B) Pearson's *r*: 0.80

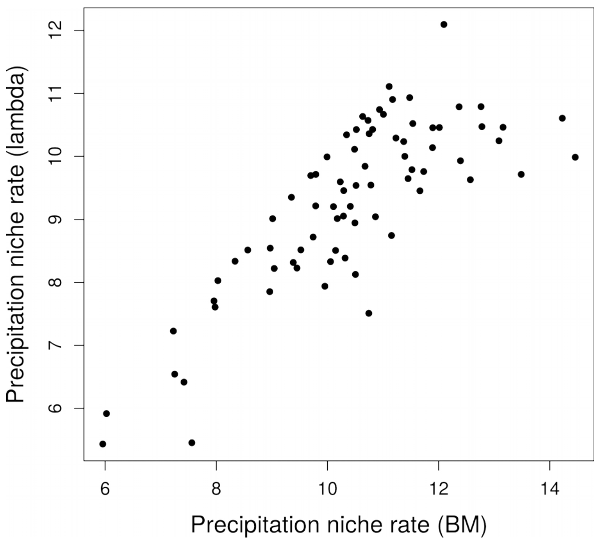

Appendix S7. Comparison of temperature and precipitation niche evolution rates obtained using phylogenies of 18 mammal families from two different sources. The second to fourth columns show the number of species in the phylogeny used for the results in the main text (from Rolland et al. 2014), number of species in alternative phylogenies (from Arnold et al. 2010), and the number of shared species between them. The last four columns show the values of niche evolution rates estimated from the phylogenies from both sources.

Family	Species Rolland et al. 2014	Species Arnold et al. 2010	Shared species	T niche evolution rate Rolland et al. 2014	P niche evolution rate Rolland et al. 2014	T niche evolution rate Arnold et al. 2010	P niche evolution rate Arnold et al. 2010
Hominidae	6	6	6	24.7	8209.3	39.0	8733.9
Atelidae	24	13	13	62.0	88550.7	122.9	122355.7
Callitrichidae	36	25	23	76.3	165060.5	72.3	108777.4
Cebidae	12	9	9	68.7	146403.1	24.5	132019.3
Tarsiidae	7	4	3	54.7	102327.5	87.4	8920.4
Indriidae	10	11	9	69.4	11907.2	105.0	50860.4
Lemuridae	19	18	17	295.1	69740.4	174.2	74264.9
Lorisidae	8	10	8	7.7	17017.7	91.0	220093.4
Galagidae	18	11	11	49.1	21857.6	141.0	32137.0
Rhinocerotidae	5	5	5	14.1	45733.1	7.4	19291.9
Tapiridae	4	4	4	679.9	3066.1	763.5	3207.1
Canidae	34	32	31	6068.8	235383.9	1973.7	53819.9
Procyonidae	14	10	10	523.3	43295.2	249.2	25114.8
Ursidae	8	8	8	1388.9	24523.7	1649.9	30297.0
Camelidae	3	3	3	24.6	1377.3	41.5	1571.7
Moschidae	7	5	5	710.4	48091.9	1179.5	36488.4
Suidae	18	12	12	357.9	86927.0	449.0	137591.0
Tayassuidae	3	3	3	0.9	29505.6	2.8	34653.0

Appendix S8. Results of the forward stepwise procedure used to select the most parsimonious model explaining diversification rate (estimated with different relative extinction fractions, ε) using niche position variables. The tables show the results of an ANOVA comparing the models specified in the first two columns.

Table S8.1

ε = 0.45

Step	Model	*F*	d.f.	*P*
Step 1:<br>Div. rate ~ 1 (null model)<br>vs.<br>Div. rate ~ x	x = BIO1	5.04	1, 90	0.027
	x = BIO5	0.96	1, 90	0.331
	x = BIO6	6.34	1, 90	0.014
	x = BIO12	3.46	1, 90	0.066
	x = BIO16	2.16	1, 90	0.145
	x = BIO17	3.85	1, 90	0.053
Step 2:<br>Div. rate ~ BIO6<br>vs.<br>Div. rate ~ BIO6 + x	x = BIO1	0.96	1, 89	0.329
	x = BIO5	1.42	1, 89	0.237
	x = BIO12	0.15	1, 89	0.697
	x = BIO16	0.08	1, 89	0.778
	x = BIO17	1.19	1, 89	0.279

Final model: Diversification rate ~ BIO6

Table S8.2

ε = 0.0

Step	Model	*F*	d.f.	*P*
Step 1:<br>Div. rate ~ 1 (null model)<br>vs.<br>Div. rate ~ x	x = BIO1	4.84	1, 90	0.030
	x = BIO5	0.85	1, 90	0.359
	x = BIO6	6.15	1, 90	0.015
	x = BIO12	3.53	1, 90	0.064
	x = BIO16	2.21	1, 90	0.140
	x = BIO17	3.97	1, 90	0.049
Step 2:<br>Div. rate ~ BIO6<br>vs.<br>Div. rate ~ BIO6 + x	x = BIO1	1.06	1, 89	0.307
	x = BIO5	1.59	1, 89	0.211
	x = BIO12	0.22	1, 89	0.643
	x = BIO16	0.03	1, 89	0.853
	x = BIO17	1.32	1, 89	0.254

Final model: Diversification rate ~ BIO6

Table S8.3

ε = 0.9

Step	Model	*F*	d.f.	*P*
Step 1: Div. rate ~ 1 (null model) vs. Div. rate ~ x	x = BIO1	5.53	1, 90	0.021
	x = BIO5	1.24	1, 90	0.268
	x = BIO6	6.79	1, 90	0.011
	x = BIO12	3.33	1, 90	0.071
	x = BIO16	2.06	1, 90	0.155
	x = BIO17	3.54	1, 90	0.063
Step 2: Div. rate ~ BIO6 vs. Div. rate ~ BIO6 + x	x = BIO1	0.79	1, 89	0.378
	x = BIO5	1.09	1, 89	0.299
	x = BIO12	0.05	1, 89	0.826
	x = BIO16	0.24	1, 89	0.623
	x = BIO17	0.87	1, 89	0.352

Final model: Diversification rate ~ BIO6

Appendix S9. Results of the variance partitioning on a full model of species richness with clade niche width (NW), geographic extent (GE), niche divergence (ND), and niche evolution (NE) as explanatory variables, but using clades defined with an age equal to the mean stem group age of all families (mean stem group age = 41.7 Mya). 86 clades were used in this analysis. Results are shown as percentage of explained variance. Full model: $r^2 = 0.73$, $F_{5,80} = 42.48$, $P < 0.001$.

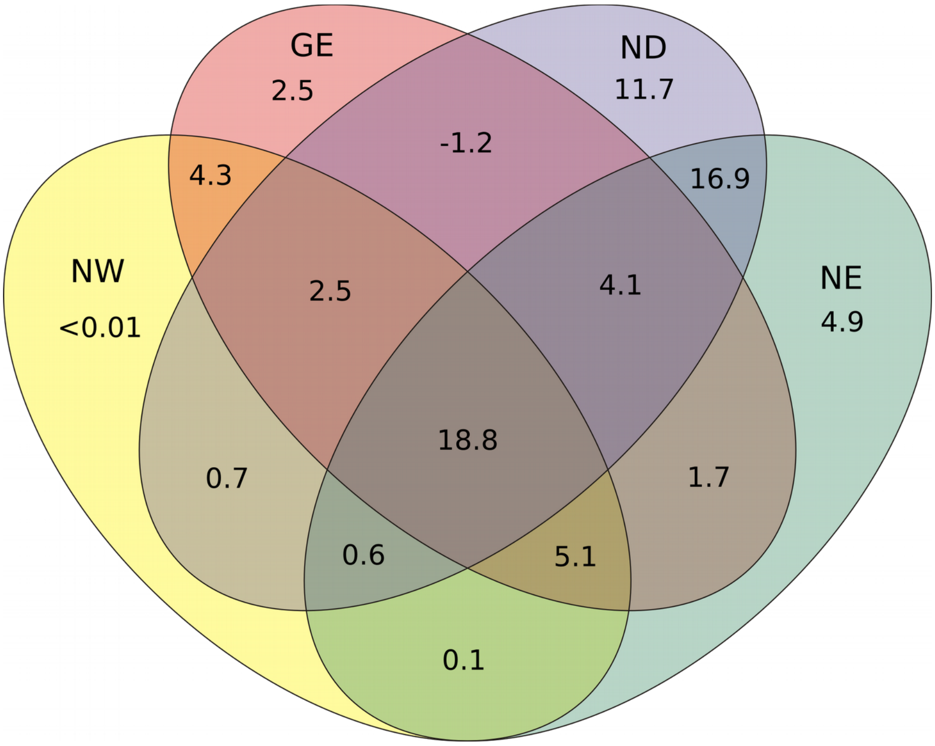

Appendix S10. Results of the PGLS between niche evolution rates and mean species niche width of each family.

Temperature niche evolution rate

$r^2 = 0.006$, $F_{1, 90} = 9.061$, $P = 0.48$

Precipitation niche evolution rate

$r^2 = 0.014$, $F_{1, 90} = 1.275$, $P = 0.26$

Appendix S11. Kernel density plots showing the distribution of r^2 across 1000 replicates of 92 randomly sampled clades of richness equivalent to real families under three different null models (unconstrained, geographically constrained, and climatically constrained). These distributions correspond to the r^2 of PGLS analyses conducted to assess the relationship between a) family niche width and mean species niche width, b) diversification rate and family niche width c) diversification rate and mean species niche width, and d) diversification rate and niche divergence. The observed r^2 is indicated with a vertical gray line.

Figure S11.1. Distribution of r^2 across 1000 replicates in the unconstrained null model (species from each null clade are sampled from the pool of all mammal species). The observed r^2 is indicated with a vertical gray line.

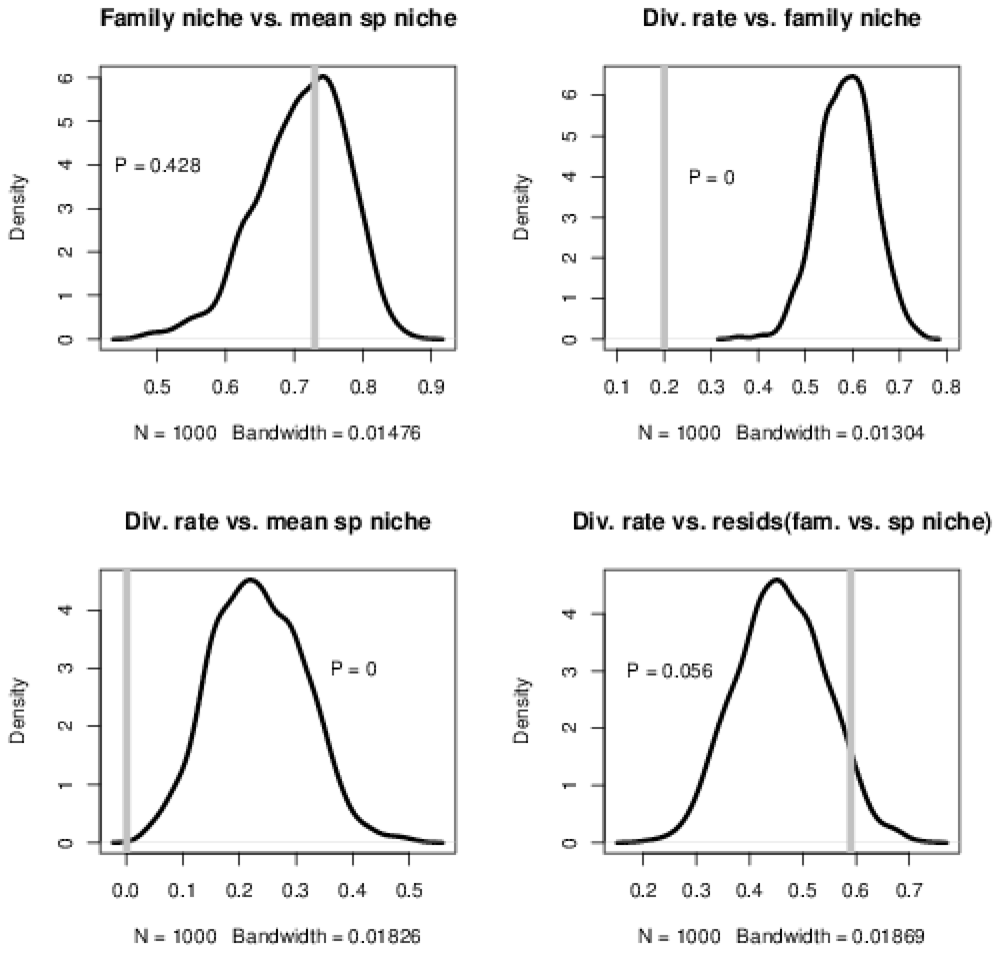

Figure S11.2. Distribution of r^2 across 1000 replicates in the geographically constrained null model (species from each null clade are sampled from the pool of species within the latitudinal and longitudinal extent of the corresponding original family). The observed r^2 is indicated with a vertical gray line.

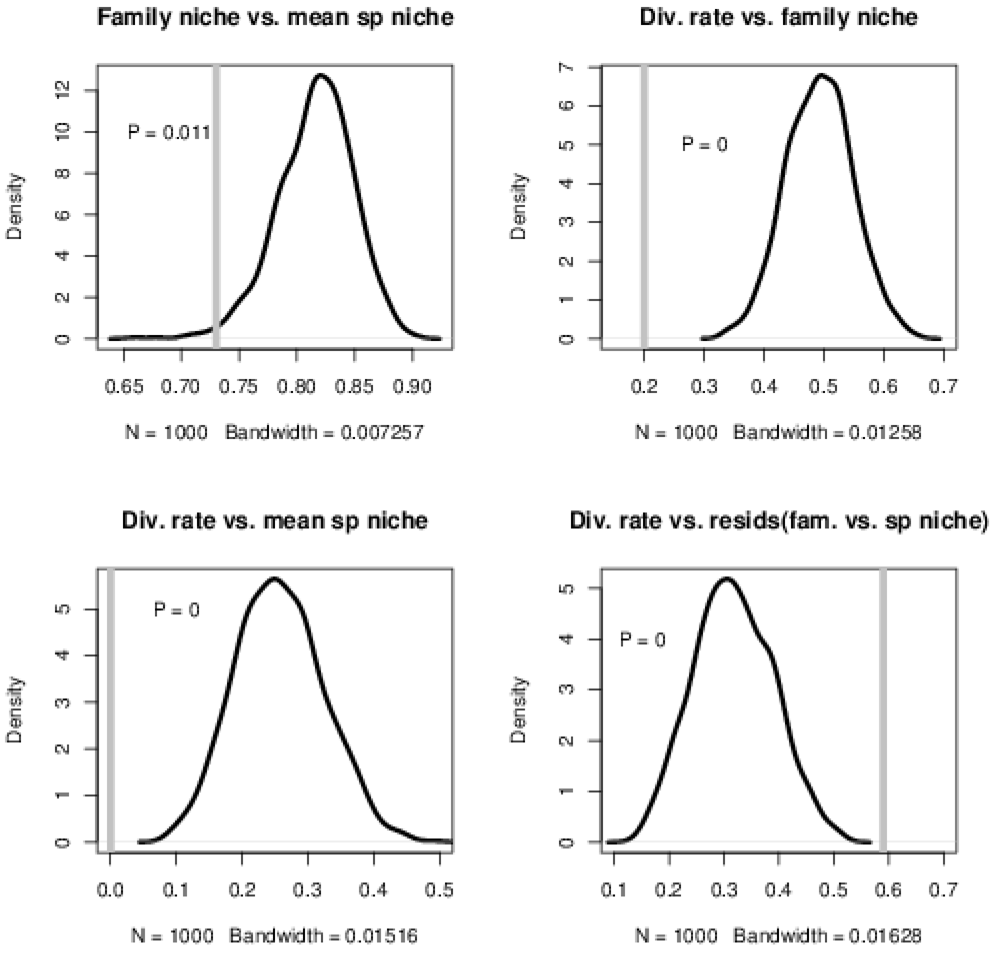

Figure S11.3. Distribution of r^2 across 1000 replicates in the climatically constrained null model (species from each null clade are sampled within the original family climatic niche). The observed r^2 is indicated with a vertical gray line.

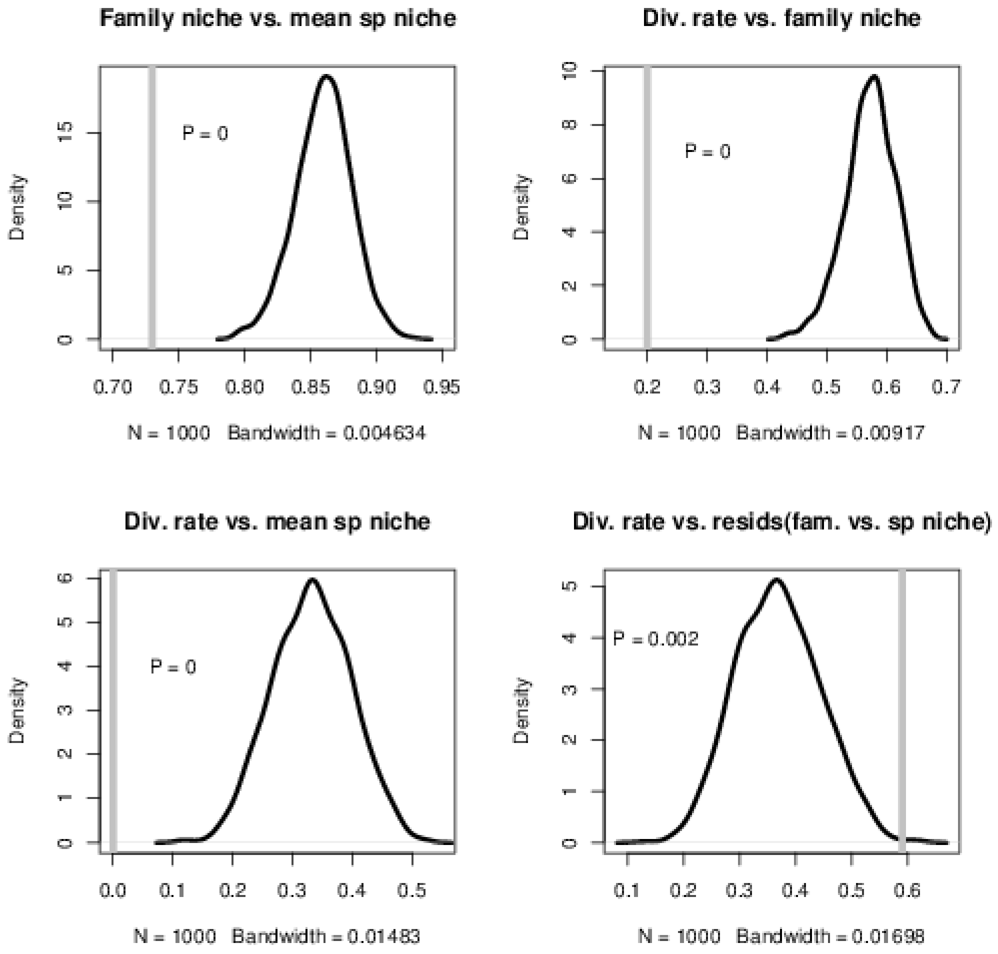

Appendix S12. Comparison of measures of family niche width. Family niche width calculated as the product of the climatic ranges on the six climatic variables (measure used in this study) is very strongly correlated with family niche width calculated multiplying the ranges on the first two principal components (explaining 97.12% of the variance in the original variables) of a PCA on the six climatic variables (Pearson's $r = 0.937$).

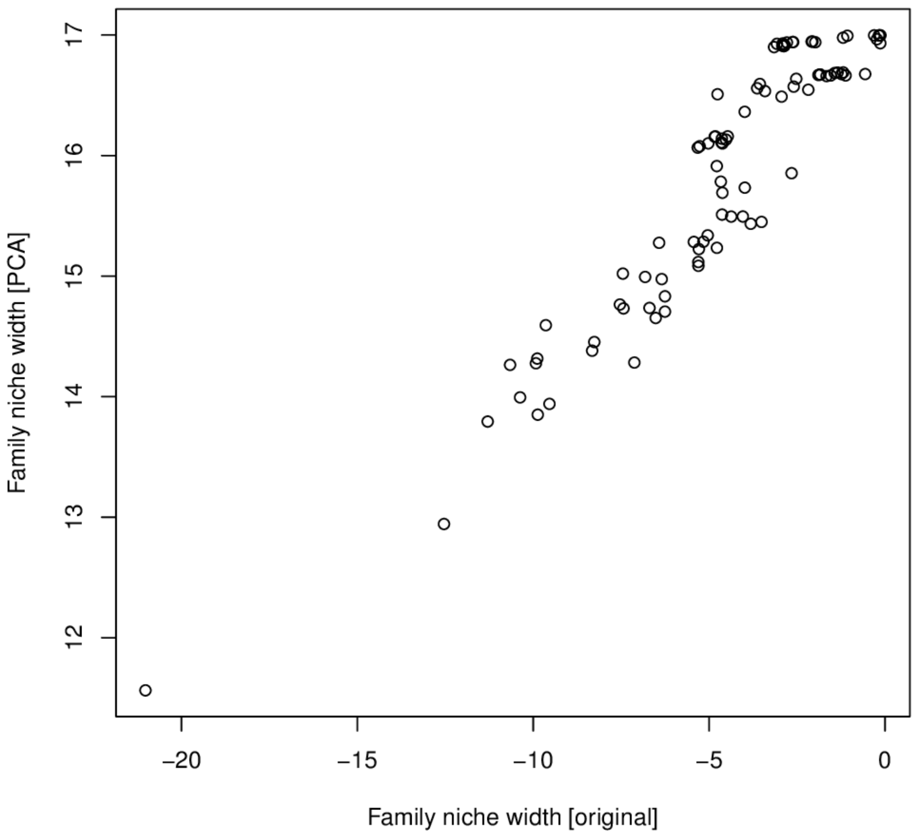

Supplement: Supplementary file 1 — Supplementary Material [file 41598_2018_27068_MOESM1_ESM.pdf]
